# Human *O-*GlcNAcase catalytic-stalk dimer anchors flexible histone binding domains

**DOI:** 10.1038/s42004-025-01813-7

**Published:** 2025-12-09

**Authors:** Sarah B. Nyenhuis, Agata Steenackers, Mana Mohan Mukherjee, Jenny E. Hinshaw, John A. Hanover

**Affiliations:** 1 Laboratory of Molecular Biology, NIDDK, National Institutes of Health, Bethesda, MD USA; 2 Laboratory of Cell and Molecular Biology, NIDDK, National Institutes of Health, Bethesda, MD USA; 3 Present Address: Versiti Blood Research Institute, Milwaukee, WI USA

**Keywords:** Cryoelectron microscopy, Glycobiology, Enzymes

## Abstract

Although thousands of proteins are specifically *O-*GlcNAc modified, the molecular features recognized by the enzymes of *O-*GlcNAc cycling (OGT/OGA) remain poorly defined. Here we solved the structure of the long isoform of human OGA (OGA-L) by cryo-electron microscopy (cryo-EM) providing a physiologically relevant platform to study the enzyme. The catalytic-stalk dimer structure was solved to a resolution of 3.63 Å, and the locally refined OGA A- and B-chains to 2.98 Å and 3.05 Å respectively. Intriguingly, the cryo-EM structures also exhibit lower resolution densities associated with the pHAT domains, suggesting substantial flexion of these domains relative to the catalytic-stalk dimer. OGA-L binds to a small subset of the 384 modified histone tails on a commercial histone peptide array. High affinity binding of OGA-L was detected to recombinant DNA-containing mononucleosomes bearing the H3K36^Me3^ and H4K^5,8,12,16Ac^ modifications. The OGA-L–H3K36^Me3^ interaction was further validated by traditional ChIP experiments in MEFs. Thus, OGA-L binds to two modified histone tails of nucleosomes linked to open chromatin, whereas it does not bind to marks associated with repressive chromatin. This model is consistent with OGA-L acting as a ‘reader’ of histone modifications linked to development, transcriptional activation, transposon silencing, and DNA damage repair.

## Abstract (web summary)

Despite the widespread occurrence of *O*-GlcNAc modifications on proteins, the specific molecular interactions of *O*-GlcNAc cycling enzymes remain elusive. Here, the authors use cryo-electron microscopy to resolve the structure of human *O-*GlcNAcase (OGA-L) as a catalytic-stalk dimer, revealing its binding to modified histone tails.

## Introduction

The *O-*GlcNAc transferase (OGT) enzyme catalyzes the addition of *O-*GlcNAc to target proteins, while *O-*GlcNAcase (OGA) removes this modification. These two enzymes have become important subjects of research following the discovery of *O-*GlcNAc modification and its crucial role as a post-translational modification in regulating protein activity, localization, and degradation^1–5^. Furthermore, *O-*GlcNAcylation is integral to many cellular signaling pathways, and its dysregulation has been linked to conditions such as insulin resistance, diabetes, cancer, lupus, and neurodegenerative diseases^3,4,6–9^. In humans, mutations in OGT are associated with X-linked intellectual disability, with alterations in OGA levels observed in some of these patients^5,10–14^. The enzymes of O-GlcNAc cycling are also coordinately regulated by a detained intron retention mechanism, which senses O-GlcNAc levels leading to alternative splicing^15^. *O-*GlcNAcylation targets serine and threonine residues on thousands of intracellular proteins and shows considerable crosstalk with kinase cascades and phosphorylation^3,8,16^. Unlike phosphorylation, which is regulated by numerous kinases and phosphatases, *O-*GlcNAcylation is mediated by only two enzymes: OGT and OGA. Despite the well-documented importance of OGT and OGA, the molecular mechanisms underlying substrate recognition by these enzymes remain poorly understood. A significant challenge has been the absence of structural information, particularly for the full-length forms of these proteins. Human OGA (hOGA) is located on chromosome 10, with two major isoforms: OGA-L (long), a 103 kDa nucleocytoplasmic protein, and OGA-S (short), a 75 kDa nuclear protein associated with nascent lipid droplets^4,17^. The N-terminal domain of OGA forms an alpha-beta barrel with hydrolase activity, while the stalk domain, composed of alpha-helices, contributes to OGA dimerization, as revealed in recent structural studies^18–20^. The longer isoform differs from the short isoform in containing a “pseudo-HAT” domain with a predicted fold like MYST domain histone acetyltransferases^21^. This domain of the protein lacks the ability to bind Acetyl-CoA or catalyze the acetyl transfer reaction. The C-terminal pseudo-histone acetyltransferase domain (pHAT) remains structurally uncharacterized in mammalian OGA^21^. Notably, the constructs used in the latest structural studies included the catalytic domain and part of the stalk domain, with some regions replaced by linkers, but lacked the C-terminal pHAT domain. To date, the full-length structure of OGA is unavailable, and the role of the pHAT domain in substrate recognition and enzyme function is unclear.

In addition to key roles in cytoplasmic signaling, *O-*GlcNAc cycling is an important contributor to epigenetic programming and the regulation of gene expression^2,5,22–28^. In mammals, both OGT and OGA are essential for normal development^24,29^. Both OGT and OGA are involved in processes linked to post-translational alterations in histones and higher order chromatin structures such as polycomb repression, transcriptional activation, and DNA damage repair^24,30–35^. The direct *O-*GlcNAc modification of histones themselves has been somewhat controversial^36,37^, but the interconnections between the histone code and *O-*GlcNAcylation are clear from genetic and biochemical studies^2,30,38,39^. Notably, OGT is associated with the polycomb complex PRC2 governing H3K27 methylation^24,30–33,38^. OGT is required for polycomb repression in the well-studied *Drosophila* system^32,33^. In this system, loss of OGA leads to a spread of polycomb repression at different sites in the genome^30^ and transcriptional deregulation^22,30^. In addition, OGA plays a role in transcriptional activation associated with activating histone modifications such as H3K36 methylation and acetylation^22,27,30,40,41^. Despite the well-documented importance of OGT and OGA in regulating gene expression, the molecular mechanisms underlying substrate recognition by these enzymes remain poorly understood. Understanding how the structure of the enzymes of *O-*GlcNAc cycling contributes to these biological functions is an important challenge. The gene encoding OGT in humans is on the X chromosome and is subject to X-inactivation in females, making its role in transcriptional repression particularly interesting^24,42–44^. The structure of the OGT protein has been studied extensively by crystallography and cryo-electron microscopy (cryo-EM), leading to a model of catalysis, dimerization, and substrate recognition^45–50^. In addition, one of these recent studies suggested how OGT and OGA may interact structurally to produce an autoinhibitory complex^45^.

Here we solved the cryo-EM structure of full-length hOGA, where the catalytic-stalk dimer resolved to 3.63 Å, and locally refined OGA A-chain to 2.98 Å and OGA B-chain to 3.05 Å, while due to flexibility, the density associated with the pHAT domain is lower resolution. The structure reveals that the catalytic core is similar to the crystal structures but differs slightly in the dimer interface. The C-terminal pHAT domain is connected by an intrinsically disordered region, facilitating multiple orientations. To gain insight into the function of domains of the OGA, we performed biochemical, enzymatic, and structural studies on the individual domains and the intact OGA-L. Both OGA-L and OGA-S show enzymatic activity towards model substrates. The presence of the pHAT domain was shown to influence OGA enzymatic activity. Histone arrays experiments, interrogating 384 combinations of modified histone peptides using the intact molecule and individual domains, suggest that the pHAT domain and OGA-L bind to a limited subset of histone tails: H3K36^Me1–3^ and acetylated H4 peptides. We confirmed these findings by demonstrating that OGA-L binds to H3K36^Me3^ and H4K^5,8,12,16Ac^ modified recombinant nucleosomes. OGA-L was also shown to interact with H3K36^Me3^ in traditional ChIP experiments in mouse embryonic fibroblasts (MEFs). Based on these structural and biochemical findings, we propose that OGA may stably recognize peptide targets, including histone tails in nucleosomes, with its pHAT domain, while the intrinsically disordered linker region allows flexibility for the catalytic-stalk dimer to recognize and deglycosylate adjacent targets up to ~ 100 Å.

## Results

### Cryo-EM structure of OGA-L

**Fig. 1:**
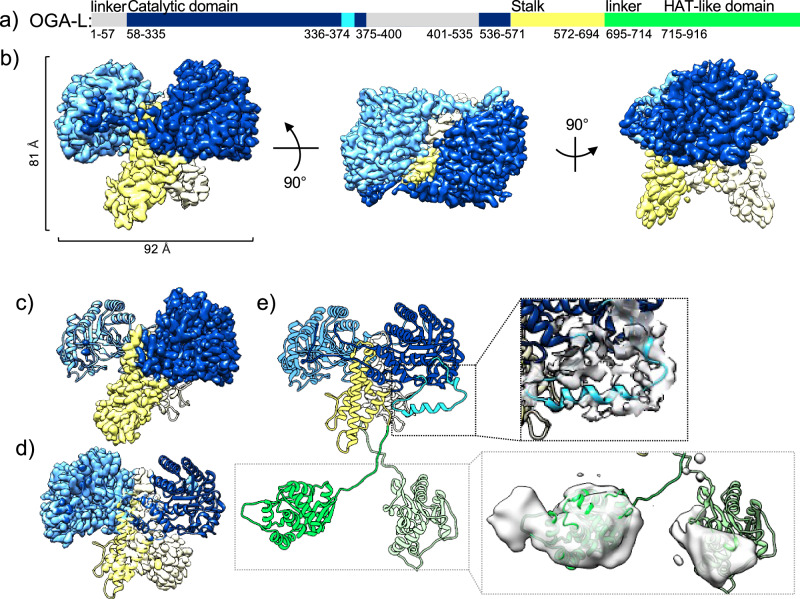
Cryo-EM structure of the OGA-L isoform. **a** Sequence diagram of OGA-L colored by domain, highlighting flexible regions. OGA catalytic domain: dark blue, unstructured regions: gray, flexible helix: cyan; stalk: yellow; linker and HAT-like domain: green. **b** cryo-EM map of OGA catalytic-stalk dimer and stalk domains colored as in (**a**), with dimensions of the dimer highlighted. cryo-EM maps of locally refined OGA A-chain (**c**) and B-chain (**d**) with catalytic and stalk domains colored as in (**b**). **e** A model of the resolved and partially resolved regions of OGA-L (left) and the density of regions that are resolved to low resolution is shown (light gray) (right). The flexible helix in the catalytic domain is highlighted in cyan, and a model of the flexible pHAT domains (green) is shown fitted into low-resolution density.

Here we present a 3.63 Å cryo-EM structure of full-length, human OGA-L in the apo state exhibiting the canonical interlocking dimer interface comprising the catalytic (blue) and stalk (yellow) domains (Fig. 1a,b, Supplementary Fig. 1). The resolution was further improved with local refinement focused on the monomers^51,52^ to 2.98 Å for OGA A-chain (Fig. 1c, dark blue) and 3.05 Å for OGA B-chain (Fig. 1d, light blue). This allowed us to build an atomic model of the catalytic-stalk dimer (Fig. 1c, d). A representative fit of the model to the map, highlighting residues 113–124 in the catalytic domain, map is shown in Supplementary Fig. 1c, and the interfacial residues in the dimer interface are highlighted as sticks with hydrogen bonding residues (orange) in Supplementary Fig. 1d and Supplementary Table 1. The buried surface area of the dimer interface is 4203.3 Å^2^ ^53^. The catalytic-stalk dimer exhibits a minor asymmetry with a displaced helix in the A-chain (Fig. 1e, cyan). Comparison between our OGA-L cryo-EM structure with existing truncated crystal structures suggests that inclusion of the flexible regions within the catalytic-stalk dimer and C-terminal pHAT region generates notable differences in the observed dimer interface (Supplementary Figs. 2 and 3, Supplementary Movie). In the catalytic dimer interface, a translational shift of ~5 Å is observed between the monomers that leads to a slight opening of the dimer by 3 Å (Supplementary Fig. 3). Removal of the flexible regions in the truncated constructs used for the crystal structures likely explains the difference observed in the dimer interface. As may be expected, AlphaFold2 structural predictions of the OGA-L better agree with our OGA-L cryo-EM structure than the truncated OGA crystal structures.

Comparing the catalytic active site between our cryo-EM model of OGA-L (blue) with eleven crystal structures (white) and one previous cryo-EM structure revealed only small differences (Supplementary Fig. 2a–n; Supplementary Table 2). This is apparent with both apo and ligand-bound crystal structures. The existing apo crystal structures (5m7r, 5vvo, 5uhk) showed little deviation or evidence of side-chain rearrangement compared to the cryo-EM map (Supplementary Fig. 2a–c, m). We also compared our structure to crystal structures with the inhibitors Thiamet G bound (5m7s,5un9, 5uhl), and PugNAc bound (5m7t, 5uho), as well as the S-linked CKII peptide target (8p01) (Supplementary Fig. 2d–i, n). The relative position of sidechains in the active site also showed little movement or rearrangement. In addition, we compared the cryo-EM map to crystal structures incorporating the inhibitors Ceperognastat (9ba8) and Aminothazole-1 (9ba9) (Supplementary Fig. 2j, k). Here, there was substantial variation of the overall dimeric structure, but little deviation in the active site. Finally, a recent cryo-EM structure of the OGA monomer in complex with OGT (7yeh) also fits well into our cryo-EM structure, with sidechains in the resolved active site positioned similarly in both structures. In each case, the sidechains visible in our cryo-EM map of the active site conform closely with the positions of residues in the active site of all crystal structures (Supplementary Fig. 2a–k, m, n) and the one cryo-EM model (Supplementary Fig. 2l).

**Fig. 2:**
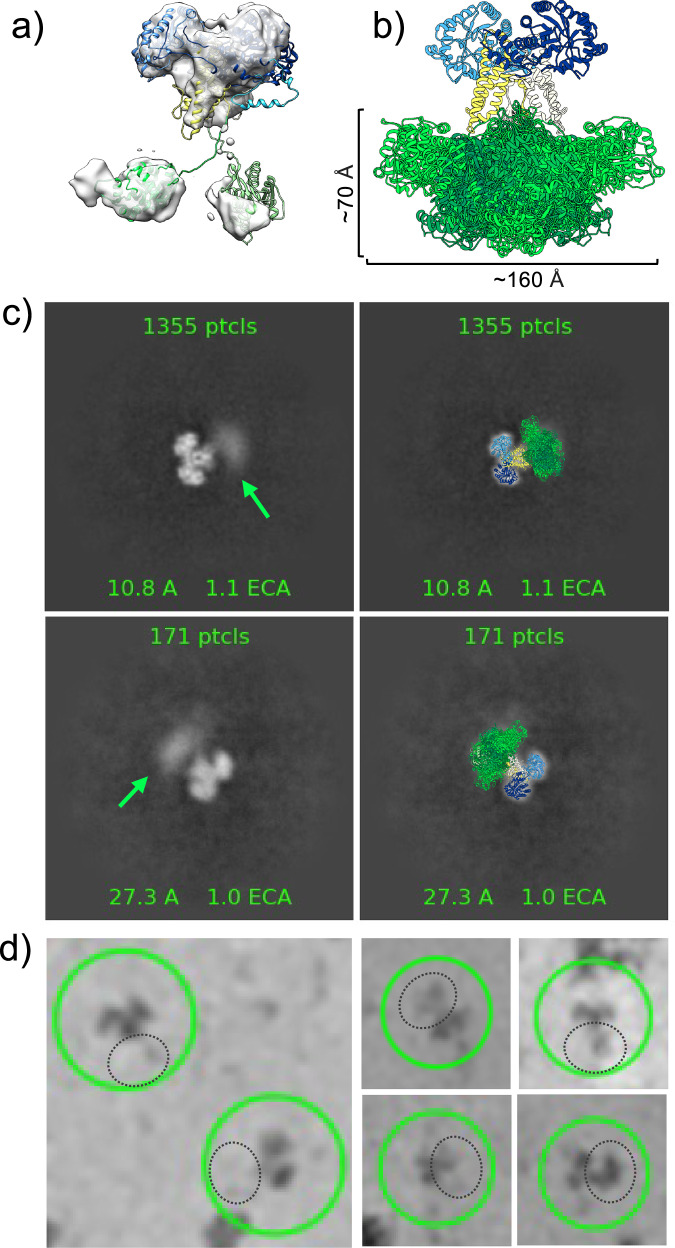
Flexibility of the pHAT domain. **a** A low-resolution cryo-EM map reveals density in the region of the pHAT domain. A representative model, colored by domain: catalytic domain, dark blue; stalk, yellow; linker and pHAT domain: green shades, is fit into the map. **b** Model colored as in (**a**). An ensemble of pHAT domains produced by the FastFloppyTail application in Rosetta^52^. Dimensions were measured for the ensemble and 2D classes produced in cryoSPARC^49,50^. **c** Left, 2D class averages of OGA exhibit clouds of density, below the catalytic-stalk dimer, that are consistent with flexible pHAT domains (green arrow). Right, representative models of OGA, colored as in (**a**), overlaid with the 2D class averages. **d** Single particles of OGA showing different positions of the pHAT domains produced from manual selection in cryoSPARC. OGA particles are circled (green) with potential pHAT domains indicated by dashed black circles.

In our high-resolution OGA structure, there is a lack of density associated with the C-terminal region of the protein. However, extensive 3D classification yielded a low-resolution map with additional density that likely represents the C-terminal pHAT domains of the OGA-L dimer (Figs. 1e and 2a). In addition, expanded 2D classification of OGA, with fewer particles in each class, also revealed clouds of density below the catalytic-stalk dimer (Fig. 2c, green arrows) and in individual particles (Fig. 2d, dashed black circles). This diffuse density was separated from the rigid dimer structure in the model by a distance consistent with flexible pHAT domains. We interpret the lack of a single stable density associated with the C-terminal pHAT domain in these 2D classes to reflect the extreme conformational flexibility of the segment joining the domains. This work provided a framework to build a model of the full-length OGA-L from a combination of our high-resolution cryo-EM model of the catalytic-stalk dimer and an Alphafold2 prediction of the pHAT domains docked into the low-resolution density (green) (Fig. 1e). In this model, the pHAT domains are defined, and the densities correspond to the size and fold predicted from Alphafold2 and homologous crystal structures^21^. However, the resolution was insufficient to orient the Alphafold2 predicted pHAT domains into the density of the map. Overall, the model provides strong evidence for a highly flexible linkage between the pHAT domains and the rigid arm-in-arm dimer comprising the catalytic and stalk domains.

Alphafold2 predictions and our cryo-EM map show a highly conformationally flexible structure. To further evaluate the flexibility of the OGA-L C-terminal domain, the program FastFloppyTail^54^ was used to produce an unbiased ensemble of models of OGA-L (Fig. 2b, green). The dimensions of the distribution of the pHAT domains (Fig. 2b) and the low-resolution OGA-L model (Fig. 2a) correlate well with the diffuse cloud of densities that span a cone-shaped area of approximately 70 Å by 160 Å (Fig. 2b, c).

It is well established that the enzymatic activity of OGA-S is significantly attenuated compared to OGA-L (Supplementary Fig. 1a, right)^55,56^. The pHAT domain clearly influences the enzymatic activity of the catalytic domain. The enzyme activity of several OGA constructs (Supplementary Fig. 4a–c) show an OGA-L mutant lacking the pHAT and linker (residues 696-917) has enhanced specific enzyme activity of OGA-L while OGA-S shows only roughly 5% of OGA-L activity (Supplementary Fig. 4d). To explore possible structural basis for this difference we compared our combined model of OGA-L (Supplementary Fig. 4e) with the Alphafold2 predicted structures of the 696-917 deletion mutant (Supplementary Fig. 4f) and OGA-S (Supplementary Fig. 4g). Overall, the catalytic and stalk regions of OGA-L and OGA-S are very similar, however OGA-S lacks a helix present in the stalk region of OGA-L that interacts in trans between the two monomers (Supplementary Fig. 4f, purple). In addition, OGA-S terminates in a short alpha-helical segment that could alter stalk movement (Supplementary Fig. 4g, orange). Considering the 696-917 deletion mutant (Supplementary Fig. 4f) has substantially elevated activity, the disordered linkers to the pHAT domains may contribute to modulating the enzymatic activity of OGA-L by acting through the helical segment in the stalk domain (Supplementary Fig. 4f, purple). Other annotated OGA isoforms (OGA-3 and OGA-4) lack segments of the OGA-L structure and are expected to alter the linkage between the pHAT domain and the catalytic-stalk domain (Supplementary Fig. 5). Notably, both OGA-3 and -4 lack residues associated with the displaced α-helix (cyan, Supplementary Fig. 5). These isoforms have deletions of exons that are alternatively spliced in O-GlcNAc depletion or elevation environments and may have a key regulatory role^15^.

### Histone-binding features of *O-*GlcNAcase and purified pHAT domains

**Fig. 3:**
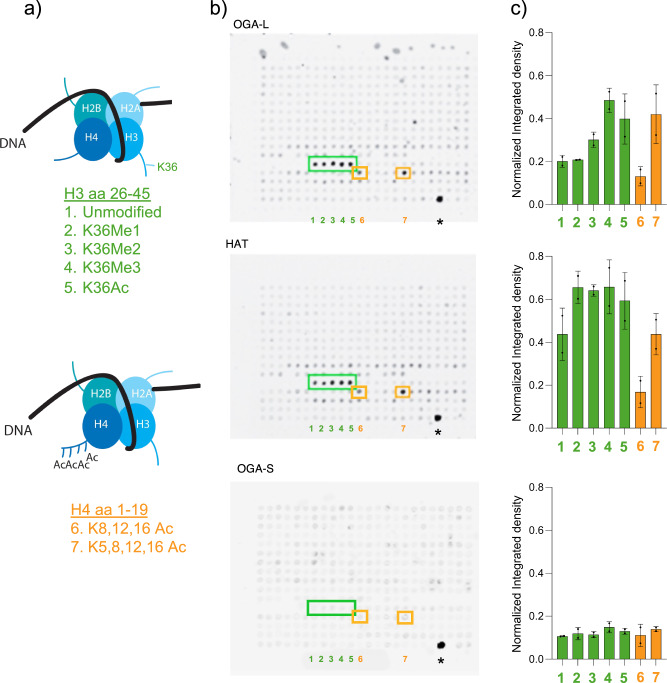
OGA-L and pHAT domains bind to a shared subset of modified histones on a modified histone array. **a** Histones showing binding to the pHAT domain and OGA-L. **b** Representative array result used for analysis; arrows indicate the positive controls for the histone array showing the maximum signal possible by this detection method. The arrays were probed and processed as described in materials and methods, and the peptides that bound to OGA-L and pHAT domains most selectively are highlighted in green and orange boxes. As shown in the lower array, little binding was observed with OGA-S lacking the pHAT domain. **c** Quantification of this representative array for OGA-L, pHAT, and OGA-S. The numbers correspond to the modified histones highlighted in (**a**, **b**). A more sophisticated analysis of the array data using the proprietary software included with the arrays is shown in Supplementary Fig. 7.

The mammalian OGA-L was previously suggested to exhibit histone acetyl transferase when purified from HeLa extracts and was termed NCOAT for Nuclear Cytoplasmic OGA and Acetyltransferase^57,58^. These authors argued for an interaction with Histone H4 and acetylation through a zinc-finger motif unique to MYST domain HATs. However, other studies have demonstrated that the pHAT domain of OGA lacks important residues for binding to Acetyl-CoA and exhibits no HAT activity in vitro^21,55^. The overall structure of the pHAT domain is like that of other histone-binding domains. The similarities in the structure of the OGA pHAT domain to other protein histone acetyltransferases prompted us to examine the ability of OGA and pHAT domains to bind to an unbiased collection of modified histone tails. The OGA-S provided an excellent biologically relevant control lacking the pHAT domain. We chose the MODified™ histone peptide array, which interrogates 384 unique histone modifications in duplicate (Supplementary Table 3). The high density of modified histone tail peptides on the array allows detection of a wide range of binding affinities. Purified human OGA-L and OGA-S and isolated pHAT domain were used to probe multiple arrays (Supplementary Fig. 6). Both the OGA-L and the isolated pHAT domain reproducibly bound to a small subset of histone tails present in duplicate on the arrays (Fig. 3a, b and Supplementary Fig. 6). The data were quantified in two independent ways. First, blots were analyzed by ImageJ to give a qualitative assessment of binding (Fig. 3c). Multiple blots were also analyzed using the commercial Array Analyze Software™ (Supplementary Fig. 6c, d, Supplementary Table 3). The OGA-S lacking the pHAT domain showed no obvious binding, while both the OGA-L variant and the pHAT domain bound with highest selectivity to acetylated H4 aa 1-19 and H3K36 26-45 peptides (Fig. 3). The binding was highest for modified H3K36 variants: K36^Me1^, K36^Me2^, K36^Me3^, and K36^Ac^ (Fig. 3b, green boxes). Multiply acetylated H4K^8,12,16Ac^ and H4K^5,8,12,16Ac^ also bound to both OGA-L and the pHAT domain (Fig. 3b, orange boxes). The Array Analyze Software™ shows “selectivity factor” for binding to each array (Supplementary Table 3). It is worth noting that only histone peptides with single modifications (one amino acid) are included in the “selectivity factor” analysis. The modified H3K36 variants were the only singly modified peptides to reproducibly show a selectivity factor above a threshold of 5. The multiply acetylated H4 peptides were clearly positive but not ranked in the Array Analyze Software™. The detection of the binding was independent of the antibody used to detect the binding on multiple arrays (His6 tag or C-terminal tag; Supplementary Table 4). The overlap in observed binding interactions between the OGA-L molecule and the isolated pHAT domain was also striking, with the highest binding interactions detected being identical. These modifications on H3 and H4 are all associated with transcriptional activation and open chromatin domains. Those interactions shared by the pHAT and OGA-L domains were (in order of specificity factor): H3K36^Me3^ > H3K36^Me2^ > H3K36^Ac^ > H3K36^Me1^ > H3K36^unmodifed^ > H4 1-19 K^5,8,12,16Ac^ > H4 1-19 K^8,12,16Ac^ > H4K16^Ac^ > H3R^17Me2^ > H3R^17Citr^ > H4R^17Me2^. Intriguingly, negligible binding was observed with most peptides on the array (Supplementary Table 3), including H3K16-35 ^S28Phos^ (position K,4) associated with histone stability or H3K27^Me3^ (position K,2) modified peptides associated with transcriptional repression. These two histone modifications are generally mutually exclusive in mammalian genomes^59^.

### OGA-L binds to certain histone marks in both recombinant nucleosomes and cell lysates

**Fig. 4:**
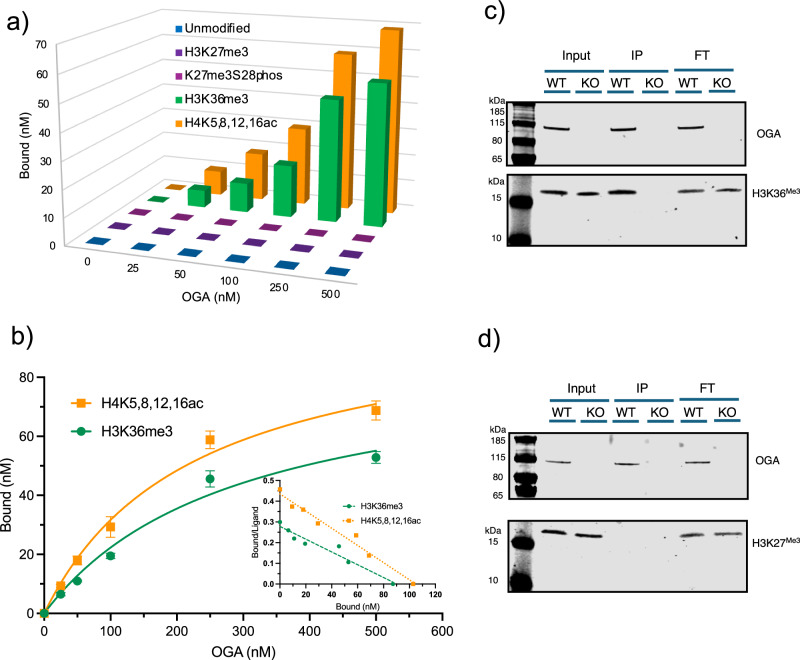
OGA-L binds to H3K36^Me3^ and H4K^5,8,12,16Ac^ modified recombinant mononucleosomes, enriching H3K36 ^Me3^ in traditional ChIP Pulldowns. **a** Saturable binding was observed with H3K36^Me3^ and H4K^5,8,12,16Ac^ mononucleosomes but not with K3K27^Me3^, K27^Me3^S28^phos^, or unmodified nucleosomes. **b** Binding parameters were determined for H3K36^Me3^ and H4K^5,8,12,16Ac^ modified nucleosomes using a saturation plot and Scatchard analysis. Nucleosomes with the H4K^5,8,12,16Ac^ modification (*K*_d_ ~ 220 nM; *B*_max_ = ~ 100 nM; Hill coefficient = 0.8), and the H3K36^Me3^ modification binds to OGA-L (*K*_d_ ~ 290 nM; *B*_max_ = ~ 90 nM; Hill coefficient = 1.9). Error bars representing the standard deviation centered on the mean. *N* = 3 independent experimental replicates. **c** Using a C-terminal anti-OGA-L, ChIP pulldown of OGA in OGA WT MEFs enriched for H3K36^Me3^ but not H3K27^Me3^. **d** Extracts from OGA KO knockout cells showed no binding to either chromatin mark. *N* = 4 independent biological replicates.

To confirm our findings suggesting that OGA-L binds to a subset of histone tails, we employed modified recombinant nucleosomes. Commercially available modified nucleosomes are reconstituted using a short segment of DNA and recombinant histones to produce mononucleosome octamers with chemically defined modifications. OGA-L binds avidly to mononucleosomes with the H4K^5,8,12,16Ac^ modification (*K*_d_ ~ 220 nM; *B*_max_ = ~100 nM; Hill coefficient = 0.8) and H3K36^Me3^ modified mononucleosomes (*K*_d_ ~ 290 nM; *B*_max_ = ~90 nM; Hill coefficient = 1.9) (Fig. 4a, b, Supplementary Figs. 7 and 8). Other modified or unmodified nucleosomes (Unmodified, H3K27^Me3^, H3K27^Me3^S28^phos^) showed little if any binding. The binding parameters are shown in detail as a saturation curve and Scatchard plot (Fig. 4b, Supplementary Figs. 7 and 8, and Supplementary Data). This analysis is the most direct method available for testing the hypothesis that the binding of OGA-L to mononucleosomes is both specific and of high affinity.

Since the H3K36 ^Me3^ mark is a very well-characterized histone modification in active chromatin^60^, we sought to determine if the OGA-L binding to H3K36 ^Me3^ could be detected in traditional ChIP pulldown experiments. We performed an immunoprecipitation assay under standard chromatin immunoprecipitation (ChIP) conditions previously used to map OGA-binding sites on chromatin^27^. Chromatin-fragmented and solubilized whole-cell lysates from OGA wild-type (WT) and OGA knockout (KO) MEFs were subjected to immunoprecipitation using a C-terminal anti-OGA antibody, which recognizes only the long isoform (L-OGA). H3K36^Me3^ was strongly enriched in the OGA-immunoprecipitated fraction (Fig. 4c, Supplementary Fig. 9a), whereas H3K27^Me3^ was not detected (Fig. 4d, Supplementary Fig. 9b). The lack of any modified histone signal in OGA knockout MEFs provided a stringent negative control validating antibody specificity, while the exclusive detection of a ~115 kDa band demonstrates the selective enrichment of the OGA-L isoform.

### The structural features of OGA-L suggest a mode of interaction with modified histone tails in nucleosomes

**Fig. 5:**
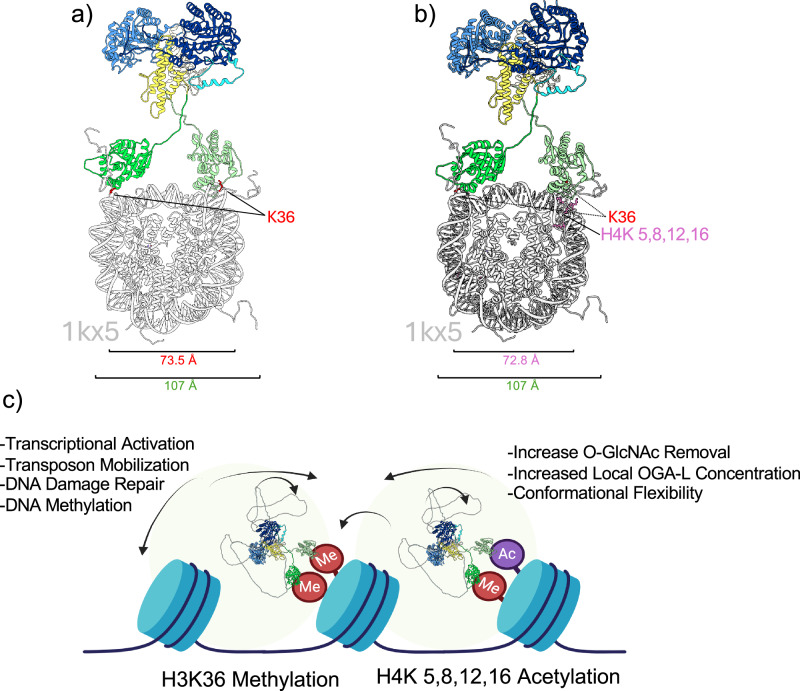
Model of OGA-L bound to histone tails of the Nucleosome. Potential binding sites of the pHAT domains to nucleosomes, highlighting proximity and spacing of the H3K36 residues (**a**) as well as the H3K36 and H4K 5,8,12, and 16 residues (**b**) in the nucleosome (PDBID:1kx5: gray). OGA is colored by domain: catalytic domain, dark blue; stalk, yellow; linker, and HAT-like domain: green. The distance between K36 residues (red) is 73.5 Å, and the total distance between pHAT densities is 107 Å (green). The distance between the K36 residue and the H4K 5,8,12,16 is 72.8 Å (pink). **c** OGA-L pHAT binding to histone modifications such as H3K36^Me^ and acetylated H4 tails would facilitate recruitment to sites of active transcription and DNA repair. The structural features identified for OGA-L are likely to increase the local concentration of tihe OGA-L and allow flexible movement of the catalytic domain to facilitate *O-*GlcNAc removal from proteins in proximity. The OGA model is shown with unstructured linkers added from the Alphafold2 colored by domain: catalytic domain, dark blue; stalk, yellow; linker, and pHAT domain, green.

The identification of the modified histones that bind to the pHAT domain and our cryo-EM structure allowed us to model OGA-L bound to nucleosomes through the pHAT domains, representing a possible physiological interaction (Fig. 5). The histone tails of the nucleosome are known to be highly flexible appendages to the core histone octamer^61^. The OGA-L cryo-EM structure is shown in juxtaposition with the position of the two H3K36 residues that are methylated or acetylated on the X-Ray structure of the nucleosome (1kx5) (Fig. 5a). Remarkably, the distance between the two modified H3K36 histone tails (~74 Å) could easily be bridged by the flexible pHAT domains in our cryo-EM structure. Similarly, the distance between the two H4K^5,8,12,16Ac^ sites on nucleosomes (~73 Å) could each be recognized by the pHAT domains in our cryo-EM model (Fig. 5b). The intrinsic flexibility of the OGA-L molecule would also allow substantial conformational flexibility of the tethered catalytic-stalk dimer upon binding to the modified nucleosomes. The potential biological implications of these findings are summarized in a model in Fig. 5c. OGA-L binding was not observed with other nucleosomes, including H3K27 methylation or H3S28Phos histone modifications (Fig. 4). These modifications correlate with closed chromatin states associated with transcriptional silencing, transposon repression, and DNA damage and methylation. In contrast, the histone modifications to which OGA-L binds are linked to open chromatin conformations. These modifications are associated with transcriptional activation, transposon mobilization, DNA damage repair, and DNA methylation/demethylation. Thus, the histone interactions observed would promote increased *O-*GlcNAc removal by increasing the local concentration of the OGA-L enzyme in open chromatin, and the conformational flexibility of the enzyme would allow *O-*GlcNAc removal from targets in proximity, such as the carboxy-terminal domain (CTD) domain of RNA Polymerase II or components of the DNA damage response. Like the 55 heptad repeats comprising the CTD domain of RNA Polymerase II, most known OGT targets are *O-*GlcNAc-modified in intrinsically disordered regions^8^.

## Discussion

### The enzymes of *O-*GlcNAc cycling are conformationally flexible multi-domain proteins

Like the TPR domain of OGT, OGA-L exhibits substantial conformational flexibility that may relate to its enzymatic and cellular functions. Although considerable progress has been made in defining the structure of these enzymes, the details of the molecular recognition of protein targets remain poorly understood. These two enzymes act antagonistically by adding and removing *O-*GlcNAc from thousands of cellular proteins and therefore are likely to have both diverse and versatile modes of target recognition^8^. The spring-like flexibility of the OGT TPR domain may provide a scaffold for binding of disordered peptides, as was observed with a recent structure of an OGT-OGA complex^45^. This structure of the dimeric OGT associated with OGA (a substrate for OGT) demonstrates that the inner portion of the TPR domain forms extensive contacts with the intrinsically disordered regions of the OGA monomer. These authors have proposed that this complex is autoinhibitory; neither enzyme is enzymatically active when associated in this fashion. It is of considerable interest to determine whether the interaction between the two enzymes of *O-*GlcNAc may be a part of their intracellular regulation. Here, we solved the structure of OGA-L by cryo-EM methods. The observed structural features of this molecule provide important insights into how it may function.

### Cryo-EM of human OGA-L

The cryo-EM structures of OGA-L reported here significantly add to existing knowledge of the organization of the enzyme (Fig. 1). Previous crystal structures were obtained by truncation of the pHAT domain and disordered regions to obtain crystal structures of the catalytic-stalk dimer. Thus, substantial segments, including the entire unstructured C-terminal domains, were excluded. Here, we used a full-length OGA molecule to generate a physiologically relevant cryo-EM map. The overall cryo-EM structure of the catalytic-stalk dimer differs from previous crystal structures in the dimer interface, where intrastrand helices hold the catalytic-stalk dimer together^18–20,62^. The cryo-EM structure also resolves the TIM-barrel structure within the catalytic center of the molecule, and the position of active site residues is not dramatically different from those observed in the crystal structures. As the crystal structures demonstrated, the catalytic domain of OGA is formed as an “arm in arm” dimer in which the catalytic domain of one monomer associates with the stalk domain of the other monomer^18–20,62^. This organization creates a substrate-binding cleft, and residues on the cleft surface could potentially afford extensive interactions with peptide substrates^18–20,62^. Our cryo-EM map of full-length OGA-L approximates the existing crystal structures, confirming this dimeric organization and the overall conformation of the molecule. However, the cryo-EM map deviates from crystal structures in several important ways. First, the presence of the additional flexible regions and pHAT domain in the OGA-L construct likely resulted in the differences observed between our cryo-EM map and crystal structures, particularly the decreased packing within the catalytic-stalk dimer interface. This may explain why Alphafold2 predictions better match the cryo-EM structure than existing crystal structures. Second, we observe an asymmetry between the two chains of the OGA-L molecule with a displaced helix visualized on one of the chains. This helical segment is positioned so that it could alter either the interaction with substrates or catalytic efficiency. Notably, this helix is partially deleted in the two OGA isoforms, OGA-3 and -4.

### OGA-L has an intrinsically disordered flexible linkage between the catalytic and histone-binding domains

The cryo-EM structure of the hOGA also provides evidence for substantial flexibility between the intrastrand dimer of the catalytic and stalk domains and the pHAT domains. The 2D classes show evidence of diffuse electron density corresponding to various positions of the histone-interacting domains (pHAT). These segments are likely to exhibit substantial flexion with respect to the stable dimer of the catalytic and stalk domains as predicted by Alphafold2. The overall dimensions suggest that the intact molecule may span roughly 70 Å from the pHAT domain to each catalytic domain. The spacing of the ordered catalytic-stalk dimer and the diffuse density corresponding to the pHAT domain is consistent with the predicted length of the intrinsically disordered linker region. Thus, OGA-L is not a rigid dimeric molecule but consists of a highly stable catalytic-stalk dimer linked to mobile histone-interacting domains by a flexible linker. This structural flexibility may have a significant effect on the functioning of the enzyme as described below.

### pHAT domain binding to a small subset of H4 and H3 histone tail modifications

Both the OGA-L and the purified pHAT domain were used to probe modified histone arrays, allowing a reproducible and unbiased assessment of histone tail interactions. This analysis demonstrated that both the pHAT domain and the OGA-L bound to only a small overlapping subset of histone tail modifications. The overlap between the observed binding with the isolated pHAT domain and OGA-L was striking. In each case, only variants of histone H3 and H4 bound substantially. No considerable binding was seen with the OGA-S lacking the pHAT domain. Little if any detectable binding was observed with the vast majority of the 384 peptides on the arrays, including H3K27 and H3K28 variants. The most considerable binding was observed with di-and tri-methylated H3K36-containing peptides. Noteworthy binding was also observed with H4K^5,8,12,16Ac^. To further support a biological relevance to the histone tail interactions, OGA-L was found to strongly bind to H3K36^Me3^ and H4K^5,8,12,16Ac^ modified recombinant nucleosomes. No binding was observed for unmodified or other modified nucleosomes as predicted from our histone array experiments. This binding to certain histone peptides suggests that OGA-L is “reader” of the histone code. Intriguingly, variants of the H4 peptide were previously shown to bind to OGA, although this was attributed to substrate recognition by a “HAT” activity^57^. Both H3K36 and H4 histone modifications, which bind to OGA-L, are associated with transcriptional activation and open poly-nucleosome conformations^59,63^. While these findings advance understanding of the underlying recognition mechanism, further experiments such as chromatin-binding assays, ChIP-seq profiling, enzymatic analyses on nucleosomal substrates, and determination of the exact point of interaction will be conducted to define how these interactions function in vivo and published in a subsequent publication.

### OGA-binding histone modifications have important roles in transcriptional activation and transposon silencing

The OGA-L is localized in both the nucleus and cytoplasm^17^. Here, we have shown that the OGA pHAT domain also binds to methylated and acetylated forms of H3K36Me^1–3^ and H4K^5,8,12,16ac^ modified histone tail peptides and recombinant nucleosomes. The small number of modified histones that bind to OGA via the pHAT domain provides an important clue as to how OGA-L functions at the level of chromatin structure.

The H3K36Me^1–3^ and H4K^5,8,12,16ac^ histone tail modifications are both thought to induce open nucleosome conformations required for active transcription^35,59^. However, they may induce these open nucleosome conformations by different mechanisms^63^. This open conformation of chromatin is associated with several important biological processes. It is also intriguing that no OGA-L binding was observed with H3K27^Me3^ or H3K^28Phos^-containing peptides or the corresponding recombinant nucleosomes. These repressive modifications are associated with transcriptional silencing during the cell cycle, X-chromosome inactivation, and the silencing of retrotransposons^23,59,64^. The H3K27^Me3^ mark is a well-established mode of transcriptional repression termed polycomb repression. OGT is associated with the polycomb repressive complex (PRC2) in *Drosophila* and in mammals^24,30–33,38^. We, and others, have shown that *O-*GlcNAc levels are very high at H3K27^Me3^ regions of the genome associated with polycomb repression^30–33,38^. In general, the H3K36^Me1–3^ modifications and H3K27^Me3^ modifications are mutually exclusive across the genome. However, in some cases, these histone marks are both present at the promoters of developmentally regulated genes termed “bivalent” promoters^64^. The antagonism between the H3K36^Me1–3^ modifications and H3K27^Me3^ modifications is a well-documented aspect of transcriptional regulation. Taken together, our finding suggests that OGA-L is capable of interacting with open chromatin enriched in H3K36Me^1–3^ and H4K5,8,12,16^Ac^. The H3K36 modification is associated with actively transcribing regions of the genome where PolII is fully engaged. Many transcription factors, including the CTD of RNA Pol II, are *O-*GlcNAc-modified and may be subject to *O-*GlcNAc removal at these actively transcribing regions. In particular, the “CTD-code” of sequential phosphorylation events leading to transcription requires *O-*GlcNAc removal from the CTD domain of Pol II^28,39,40,65,66^. Once *O-*GlcNAc is removed, phosphorylation of residues within the heptad repeats of the Pol II CTD is associated with transcriptional initiation, elongation, and termination^26,28,39,40^. We have previously suggested that this series of post-translational events may be required for proper transcriptional regulation^28,39,40,65,66^.

The transcriptional repression of retrotransposons in mammals is a complex and redundant means of maintaining genome integrity^67^. Recent evidence suggests that *O-*GlcNAc cycling is an important contributor to repression of transposable elements such as LINE-1^23^. OGT was shown to be uniquely recruited to sites of LINE-1 insertion by interacting with the Zn-finger protein KAP1 at genomic regions subject to DNA methylation. When OGA-L was artificially recruited to these elements using Cas-9 fusion, LINE-1 was actively expressed. These findings suggest that OGA-L may normally be restricted from chromatin domains established during transposon silencing, which include the H3K27^Me1–3^ modification. Together, these findings provide a structural framework that lays the groundwork for future exploration of OGA’s role in epigenetic regulation.

### *O-*GlcNAcase and DNA damage repair

The H3K36^Me1–3^ and H4K5,8,12,16^Ac^ histone tail modifications, which bind to OGA-L, are each associated with the process of DNA damage repair^35,59,68,69^. Intriguingly, OGA is also required for DNA repair^25,34,70^. We and others have shown that loss of OGA in actively growing cells triggers an increase in DNA double-strand breaks^34,70^. In addition, loss of OGA induces DNA damage signaling through ATM/ATR signaling, and CHK1/2 stabilizes OGT, creating an autoregulatory feedback loop^70^. Both OGT and OGA are normally recruited to sites of DNA damage, and many DNA repair factors are *O-*GlcNAc modified^34,70,71^. A recent paper showed that the pHAT domain of OGA was required for recruitment to sites of DNA damage^34^. These authors proposed a model by which OGA is recruited to sites of DNA damage by its pHAT domain to remove *O-*GlcNAc from molecules such as Ku70/80, mediating the process of non-homologous end joining (NHEJ). Thus, our findings regarding the pHAT domain histone-binding characteristics are consistent with this model of OGA recruitment to sites of double-strand DNA breaks.

### A model of *O-*GlcNAcase and the structure-function relationship of the pHAT domain

The conformational flexibility between the two more ordered domains of OGA-L, highlighted by the cryo-EM structure, is likely to be an important contributor to the functioning of the enzyme. The presence of the pHAT domain demonstrably influences the enzymatic activity of OGA-L. The 2D classes clearly show a distribution of the flexible pHAT domain relative to the stabilized cross-chain dimer of the catalytic domain (Fig. 2c). The intrinsic flexibility of the OGA-L may be important for the functioning of the enzyme in mediating events associated with the removal of *O-*GlcNAc in proximity to modified H3K36 and other activating histone domains. Histone octamers contain two copies of the H3K36 site, which can be modified. The crystal structure of the histone octamer (pdb:1KX5) shows that these two H3K36 sites are separated by approximately 70 Å which suggests that the highly flexible OGA-L pHAT domains could potentially bind simultaneously to the two modified H3K36 sites on the nucleosome (Fig. 4). It is also possible that other combinations of histone tail modifications could be recognized by OGA-L that did not emerge from our histone array analysis.

In summary, our findings suggest that OGA binding to modified H3K36 and H4 histone tails would allow a flexible tethering and increased local concentration of the catalytically active enzymes in chromatin regions enriched in these histone modifications (Fig. 4). These modifications are both associated with open chromatin. In contrast, OGA-L shows no detectable binding to H3K27 methylation or H3K28 phosphorylation sites associated with transcriptional repression and closed chromatin. Thus, histone domains containing these modifications would have a reduced local concentration of OGA-L relative to H3K36-containing domains. In general, domains rich in methylated H3K36 and methylated H3K27 are mutually exclusive in eukaryotic genomes, suggesting that the OGA concentrations in those regions may vary dramatically. The binding of OGA-L to H3K36^Me1–3^ is consistent with the known roles for OGA in H3K36^Me1–3^-mediated processes such as transcriptional activation, transposon regulation, and DNA damage repair. These evolutionarily conserved histone modifications play a key role in development, stem cell biology, and differentiation. Our structural work has identified OGA-L as a potential “reader” of this aspect of the histone code.

## Material and methods

### Expression and purification of epitope-tagged *O-*GlcNAcase and subdomains

#### Protein expression and purification

Each of the OGA fragments and subdomains was cloned into the pBAD/HisA plasmid in frame with the epitope tags after codon optimization by GeneART Gene synthesis services (Invitrogen). Each of the resulting plasmids was sequenced to verify the coding sequence expected. These fragments and their corresponding sizes are shown in (Supplementary Fig. 1). The protein and DNA sequences for each of the fragments are also shown in (Supplementary Fig. 1). The pBAD/HisA OGA plasmids were transformed into the TOP-10 bacterial strain, which contains the *araBAD* promoter. For each protein preparation, the following steps were carried out. On day 1, a 5 mL LB-Carb culture was grown at 37 °C overnight, shaking at 200 rpm. On day 2, the cultures were pelleted at 4700 rpm for 5 min and the supernatant removed. The pellet was resuspended in 500 mL or 1 liter of Terrific Broth (Thermofisher) with Carbenicillin added (final carbenicillin concentration is 50 μg/mL). Cultures were grown at 37 °C, shaking at 200 rpm until the OD_600_ reached 0.8 (usually about 3.5 to 4 h for 1 L culture) and cooled to room temperature for 30 min. Arabinose (final concentration of 0.02%) was added to the culture to induce protein expression, and cultures were incubated at 20 °C overnight with shaking. On day 3, the culture was centrifuged at 4700 rpm at 4 °C for 10 min and the supernatant was removed. The pellet was flash-frozen in liquid nitrogen or on dry ice. The pellets were then subjected to the lysate preparation step immediately or stored at −80 °C.

### Lysate preparation

The *E. coli* cell pellets were thawed on ice, then suspended in 25 mL of Lysis buffer. The lysis buffer consisted of 50 mM NaPhos pH 7.8, 500 mM NaCl, 10 mM imidazole, and 0.1% Tween 20 to which 0.2 mM PMSF had been added fresh. Lysate was transferred to a 50 ml conical tube. The mixture was sonicated using a flat tip 3 × 30–45 s on ice. β-mercaptoethanol (BME) was added to a final concentration of 5 mM, and the mixture was inverted several times to mix. The resulting mixture was centrifuged at 35 K rpm for 30 min–1 h at 4 °C. The supernatant was the cellular lysate containing the expressed protein. After ultracentrifugation at 100,000 × *g*, the supernatant was transferred to a 50 mL conical tube. Talon resin (Takara Bio) was added directly to the supernatant. OGA proteins were allowed to bind for at least 1 h at 4 °C with spinning or rocking. For 1-liter cultures, 2 mL of the Talon resin mixture was added. The beads were pelleted by spinning very gently at 500 rpm for 2 min at 4 °C. The unbound fraction was removed, and OGA-L or subdomains were present on the beads. Wash buffer (25 microliters) was added to the beads (50 mM NaPhos pH 7.8, 500 mM NaCl, 30 mM imidazole, 5 mM BME) and shaken to resuspend the beads. The beads were pelleted by spinning very gently at 500 rpm for 2 min at 4 °C. This was repeated 3 times. The bead and liquid slurry were applied to a 5 mL Pierce Centrifuge Column (Product #89897) and spun gently at 500 rpm for 2 min at 4 °C to remove the remaining Wash Buffer. The pellet was treated with 1 mL of Elution buffer (50 mM NaPhos pH 7.8, 500 mM NaCl, 300 mM imidazole) and rocked at 4 °C for 5 min. To collect purified OGA, remove the bottom cap, place in a fresh 15 mL conical, and spin gently at 500 rpm for 2 min at 4 °C. Elution was repeated three times to maximize protein recovery. Eluted proteins were stored at 4 °C or directly used for histone array experiments. Protein staining and Western blotting of the eluted proteins suggested that the epitope-tagged proteins corresponded to the appropriate molecular mass for each of the variants or OGA-L molecules.

### Protocol for *O-*GlcNAcase binding to modified histones using Active Motif Modified Array Labeling Kit

Histone arrays were probed with OGA-L, the pHAT domain, or the short isoform lacking the pHAT domain using a minor modification of the procedure described in the manufacturer’s protocol (Active Motif MODified™ Histone Peptide Array). All values presented are based on probing 3 or more histone arrays using the 6His epitope tag antibody (Supplementary Table 4). To verify specificity, arrays were also probed with the C-term and Xpress epitope tag antibodies (Supplementary Table 4). The C-term antibody gave results very similar to the His6 tag, but the Xpress tag antibodies bound to the histone array with less specificity and were therefore excluded from the analysis. Briefly, arrays were blocked with 3 mL AM2 Blocking Buffer provided in the Active Motif Kit at RT for 1 h with shaking. Arrays were washed three times with the supplied wash buffer. For each protein probed. 100 nM of protein was added in 3 mL of 20 mM Tris pH 7.5, 1 mM DTT, 0.5 mg/mL BSA, and incubated at RT for 1 h with shaking. Blots were washed 3× prior to antibody addition. Both Anti-His6 antibody and the Anti-C-myc antibody were added at 1:2000 in 3 mL total volume of the AM2 buffer and incubated at 4 °C overnight. After 3X washing, anti-mouse-IR-800 antibody at 1:2000 in 3 mL AM2 was added and incubated at RT for 1 h with shaking. After washing two additional times, imaging was performed using the LI-COR Odyssey DLx I LICORbio Imaging System. Arrays were analyzed using the Active Motif Array Analyze Software as described by the manufacturer (Active Motif). The software program analyzes the intensity of spot interactions from the MODified Histone peptide array and generates a graphical analysis of the histone peptide modification interactions. With the Array Analyze Software, information about spot intensity, averages, and errors was saved in Microsoft Excel-compatible files, allowing for our individual analysis. Output from the Active Motif Array Software is presented as “Specificity Factors.” This is a background-subtracted graph of the 10 modifications with the greatest specificity factors. While all modifications have been accounted for in determining the specificity factor, only singly modified peptides representing the 10 modifications with the highest specificity factor values are graphed. After three such experiments, only the histone modifications observed in all three experiments are reported. For both the pHAT domain and OGA-L, 9 of the 10 identified histone modifications were found to be shared. Thus, the analysis is a very conservative estimate of the reproducible binding events. In addition, blots were quantified from the original images using ImageJ to confirm the results obtained by the Motif Array Software.

### Binding of OGA-L to recombinant nucleosomes

To a final concentration of 500 nM of the biotinylated mononucleosomes (EpicCypher, see Supplementary Table 5 for details) in 50 μL of the OGA assay buffer, various amounts of full-length recombinant OGA (0, 25, 50, 100, 250, and 500 nM) were added, and incubated overnight at 4 °C with gentle rocking. Following incubation, Pierce streptavidin magnetic beads (ThermoFisher Scientific, 88817) were used to pull down protein bound to the biotinylated mononucleosomes. Prior to use, the streptavidin magnetic beads were prewashed with binding and wash buffer (1XTBSTw) in a 1.5 ml microcentrifuge tube. Biotinylated mononucleosomes attached proteins were added to the 1.5 ml microcentrifuge tube containing prewashed magnetic beads and incubated for 1 h at room temperature with gentle rocking. After incubation, the magnetic beads were collected with a magnetic stand, washed twice with 1XTBSTw, and finally once with 0.1% Tween 20 water. Finally, bound proteins were eluted by boiling in LDS sample loading buffer. The eluted protein was subsequently analyzed by immunoblotting using anti-MGEA5 (Benthyl Laboratories, A304-345A) and visualized using the Odyssey Licor imager to detect and quantify OGA-L binding. Analysis of kinetic binding data was performed using Prism (Graphpad; Dotmatics).

### Immunoprecipitation of MEF cell lysate using a C-terminal OGA-L antibody

MEF cells were cultured in DMEM (Gibco, 10567022) supplemented with 10% (for HeLa) or 15% (for MEFs) fetal bovine serum, penicillin (100 U/mL), and streptomycin (1 mg/mL). HeLa cells were kept at 37 °C in a humidified incubator under 5% carbon dioxide (CO_2_) atmosphere, and MEFs were kept at 37 °C in an incubator with 3% oxygen (O_2_) atmosphere. Cells were seeded at a density of 500k cells in a 10 cm plate. After the appropriate time, cells were harvested by physical scraping, counted, and centrifuged at 10,000 × *g* for 5 min in 1.5 mL sterile RNAse and DNAse-free Eppendorf tubes. The supernatants were removed carefully, and the collected cell pellets were stored at −80 °C for future use.

Chromatin fragmentation and solubilization were performed based on the ChipSeq sample preparation protocol, following the procedure described elsewhere^27^. Approximately 5 × 10^6^ cells were washed twice with cold PBS and cross-linked in 1% formaldehyde for 10 min and quenched with 0.5 mL of 2.5 (M) glycine. Cells were washed twice with cold PBS and placed in 1 ml of low salt buffer (10 mM Tris-HCl, pH 7.5, 200 mM NaCl, 1 mM MgCl, 5 mM EDTA, 1 mM EGTA, 1% NP-40, 1% Triton X-100) with complete protease-phosphatase inhibitor mixture and 0.1% Benzonase nuclease (Sigma Aldrich, E1014-5KU). Cells were sonicated for 15 min (10 s on, and 30 s off) with a probe Sonicator Ultrasonic Processor XL. Lysate was spun down at 14,000 rpm for 30 min, and the supernatant was collected for immunoprecipitation using Dynabeads protein immunoprecipitation kit (Thermo Fisher Scientific, 10007D) following the manufacturer’s protocol. Briefly, 50 μL of resuspended dynabeads were prewashed thrice with 1X PBS and incubated overnight with 10 μg of Anti OGA antibody (SAB4200311, Sigma Aldrich) diluted in 200 μL of Ab Binding and Washing Buffer at 4 °C with gentle rocking. After incubation, the magnetic dynabeads were collected with a magnetic stand, supernatant was collected as flowthrough (FT) and washed twice with Washing Buffer. Finally, bound proteins were eluted by boiling with 20 μL Elution Buffer containing 10 μL LDS sample loading buffer for 10 min and ran on a western blot. The eluted protein, along with input and FT, was subsequently analyzed by immunoblotting using anti-OGA antibody (Santa Cruz, sc-376429) and anti-H3K36^Me3^ antibody (Active motif, 61022) or anti-Histone H3K27^Me3^ antibody (Active motif, 39055) to detect OGA-L-bound chromatin.

### Expression and purification of human OGA-L protein for cryo-EM

All constructs were codon optimized for expression in *E. coli*. The full-length of human OGA protein (OGA-L) was subcloned into a pBAD vector with an N-terminal His6 tag as described above. The plasmids were transformed into Escherichia coli TOP10 cells. Cultures were grown at 37 °C in Luria–Bertani (LB) medium until the optical density at 600 nm (OD600) reached 0.8. At this point, the culture was cooled to 16 °C and protein expression was induced with 0.4 mM arabinose. After 16 h of induction, cells were harvested and lysed at 4 °C using an ultra-high-pressure cell disruptor. The lysate was subjected to affinity purification on Cytiva HisTrap columns, with the target protein eluted in a single step using a buffer containing 250 mM imidazole. The protein was further purified by size-exclusion chromatography (Enrich SEC 650 10/300 MM, Bio-Rad) in a buffer containing 20 mM Tris, 150 mM NaCl, and 0.5 mM Tris(hydroxymethyl)phosphine (THP). The purified protein samples were flash-frozen in liquid nitrogen and stored at −80 °C. The resulting protein was judged to be >95% pure by Coomassie brilliant blue staining.

### *O-*GlcNAcase activity assays

OGA assays on bacterially produced recombinant OGA were performed essentially as described previously^55,72^. For assays in OGA knockout (KO) MEFs (Keembiyehetty et al.^29^), cells were lysed in RIPA buffer (10 mM Tris-HCl, 150 mM NaCl, 1% Triton X-100 [v/v], 0.5% sodium deoxycholate [w/v], 0.1% sodium dodecyl sulfate [w/v], and protease inhibitors; pH 7.5). The lysates were vortexed and centrifuged at 14,000 rpm for 20 min at 4 °C. For the enzymatic activity assay, 30 μg of clarified lysate, with or without purified OGA, was added to a reaction mixture containing 200 μM fluorescein di(*N*-acetyl-β-D-glucosaminide) (FDGlcNAc) and 50 mM *N*-acetylgalactosamine (GalNAc) in 50 mM citrate/phosphate buffer (pH 6.5). Reactions were incubated in the dark at 37 °C with shaking at 100 rpm for 30 min. The reactions were quenched by the addition of sodium carbonate (Na₂CO₃) to a final concentration of 400 mM. Fluorescence was measured in 1-s intervals at the excitation wavelength of 485 nm and at the emission wavelength of 535 nm on a Wallac 1420 fluorometer (PerkinElmer Life Sciences).

### SDS-PAGE and western blot analysis

Protein lysates were mixed with Laemmli buffer and boiled at 95 °C for 5 min prior to separation by SDS-PAGE. Samples were resolved on NuPAGE 4–20% Bis-Tris gels (Invitrogen) and visualized using Coomassie blue staining or Western blotting. For Coomassie staining, the gels were incubated in PageBlue Protein Staining Solution (Invitrogen) on a rocking shaker for 1 h, followed by destaining with water for an additional hour. For Western blot analysis, proteins were transferred onto a nitrocellulose membrane and blocked for 45 min with 5% [w/v] nonfat milk in Tris-buffered saline containing 0.1% [v/v] Tween 20 (TBS-T). Membranes were incubated overnight at 4 °C with primary antibodies diluted in milk/TBS-T with gentle agitation. After primary antibody incubation, the membranes were washed three times with 10 mL of TBS-T for 10 min per wash and subsequently incubated with fluorescent-conjugated anti-mouse secondary antibodies (LI-COR) at a 1:10,000 dilution for 1 h at room temperature. Three additional washes with 10 mL of TBS-T for 10 min each were performed before imaging the blot on an Odyssey Fc imager (LI-COR).

### Cryo-electron microscopy sample preparation, data collection, processing, and refinement

Purified OGA-L protein was diluted to a concentration of approximately 0.5 μg/μl in 20 mM Tris and 150 mM NaCl. A 3 µl aliquot of the protein solution was applied to a glow-discharged grid (30 s glow discharge in a glow discharge cleaning system, Pelco easiGlow (Ted Pella, Inc.)). Following a 15-s incubation at >95% relative humidity, excess protein was removed by blotting for 5 s. The grids were then plunge-frozen into liquid ethane using a Leica EM GP2 plunge freezer (Leica Microsystems). Vitrified grids were stored in liquid nitrogen before examination using cryo-EM.

The cryo-EM data were collected using a Titan Krios G3 microscope (Thermo Fisher), operating at 300 kV, equipped with a K3 detector (Gatan). One thousand seven hundred thirty-seven images were collected at a magnification of 105,000× with a calibrated pixel size of 0.53 Å, nominal defocus range of 0.3–2.4 μm, 50 frames, and 67 e^−^/Å^2^ electron exposure per Supplementary Movie.

Images were processed using cryoSPARC v4.6.0 (Supplementary Fig. 1b)^51,52^. Images were corrected using Patch Motion correction and Patch CTF estimation in cryoSPARC^51,52^. Particles were selected using the blob picker, extracted with a 280-pixel (1.2114.2518 Å/pixel) or 440-pixel (1.2045 Å/pixel) box size, and subsequently pruned using iterative 2D classification. Initial alignment was performed using the ab initio reconstruction job. Refinement was performed using iterative heterogeneous refinements, non-uniform refinements, 3D classifications, and local refinements using C2 or C1 symmetry. Masks for local Refinements were generated in Chimera^73^.

Maps generated in cryoSPARC were post-processed using deepEMhancer to sharpen protein densities and sharpened in cryoSPARC as a cross-comparison for model building^74^.

The initial model of the OGA dimer was produced using Alphafold2 multimer, which was fit into generated maps in UCSF Chimera^73,75^. Iterative model refinement was performed using Rosetta v2021.16, Phenix v1.29.1-4487, Gaussian mixture model-based atomic model refinement in EMAN2 v2.99.66, and Coot v0.9.8.92, then assessed using MolProbity and Phenix^75–80^. The cryo-EM data collection, final refinement, and validation statistics for the model are presented in Table 1. Structural analysis, measurements, and figures were prepared in Chimera 1.15 and ChimeraX 1.2.5^73^. Interfacial area and hydrogen bonding were calculated using the PDBePISA server^53^. RMSD values were calculated in Pymol version 2.4.0 for all atoms, backbone atoms, sidechains, and Cα atoms (Supplementary Fig. 3, Supplementary Table 2^81^).

**Table 1:** Cryo-EM data collection, refinement, and validation statistics

Data collection and processing	OGA-L Catalytic Dimer (EMDB:EMD-49293, PDB ID: 9NE2)	OGA-L Catalytic Dimer A-Chain (EMDB:EMD-49294, PDB ID: 9NE4)	OGA-L Catalytic Dimer B-Chain (EMDB:EMD-49295, PDB ID: 9NE5)	OGA-L Catalytic Dimer with extra A-chain density (EMDBEMD-49297)	OGA-L Dimer (EMDB:EMD-49296)
Microscope	Titan Krios	Titan Krios	Titan Krios	Titan Krios	Titan Krios
Magnification	105,000×	105,000×	105,000×	105,000×	105,000×
Voltage (kV)	300	300	300	300	300
Electron exposure (e^−^/Å^2^)	67	67	67	67	67
Defocus range (-µm)	0.3–2.4	0.3–2.4	0.3–2.4	0.3–2.4	0.3–2.4
spherical aberration (mm)	2.7	2.7	2.7	2.7	2.7
automation software	SerialEM	SerialEM	SerialEM	SerialEM	SerialEM
Data processing software	CryoSPARC v4.6.0	CryoSPARC v4.6.0	CryoSPARC v4.6.0	CryoSPARC v4.6.0	CryoSPARC v4.6.0
Pixel size (Å)	1.2114	1.2114	1.2114	1.2114	1.2045
Symmetry imposed	C1	C1	C1	C1	C1
total number of micrographs (no.)	1737	1737	1737	1737	1737
Final particles (no.)	65,831	65,831	65,831	62,230	8527
Box Size (pixels)	280	280	280	280	440
Map resolution (Å)	3.630	2.981	3.048	3.860	10.070
FSC threshold	0.143	0.143	0.143	0.143	0.143
**Refinement**					
Initial model	Alphafold2 Multimer	Alphafold2 Multimer	Alphafold2 Multimer		
Refinement Packages	Rosetta v2021.16 Phenix v1.29.1-4487 Coot v0.9.8.92 EMAN2 v2.99.66	Rosetta v2021.16 Phenix v1.29.1-4487 Coot v0.9.8.92 EMAN2 v2.99.66	Rosetta v2021.16 Phenix v1.29.1-4487 Coot v0.9.8.92 EMAN2 v2.99.66		
Chains (no.)	2	1	1		
Model resolution (Å)	3.70	3.90	3.90		
FSC threshold	0.143	0.143	0.143		
Map CC	0.82	0.61	0.60		
*Model composition*					
Non-hydrogen atoms	7652	3826	3826		
Protein residues	936	468	468		
Ligands	0	0	0		
*B-factor*					
Protein (Å^2^, mean)	89.56	89.48	89.63		
*Validation*					
MolProbity Score	1.28	1.19	1.30		
Clashscore	5.29	4.1	5.55		
*Ramachandran plot*					
Favored (%)	98.38	98.05	98.70		
Allowed (%)	1.62	1.95	1.30		
Disallowed (%)	0.00	0.00	0.00		
Rotamers outliers (%)	0.12	0.24	0.00		
Cβ outliers (%)	0.00	0.00	0.00		
*R.M.S. deviations*					
Bond Length (Å)	0.003 (0)	0.003 (0)	0.004 (0)		
Bond Length (°)	0.503 (1)	0.511 (1)	0.908 (4)		
*Peptide plane (%)*					
Cis proline/general (%)	4.0/0.0	4.0/0.0	4.0/0.0		
twisted proline/general (%)	0.0/0.0	0.0/0.0	0.0/0.0		
CaBLAM outliers (%)	0.99	0.88	1.10		

Motion of the disordered region, residues 694–712 (PIDGANDLFFQPPPLTPTS), between the stalk and pHAT domain was modeled using the PyRosetta-based algorithm, Fast^54^. Six hundred structures were generated, and 75 structures were used to display the ensemble in Fig. 2b. From this unbiased distribution, subsets of this ensemble were selected that best fit into the 2D class densities in Fig. 2c.

### Reporting summary

Further information on research design is available in the Nature Portfolio Reporting Summary linked to this article.

## Supplementary information

Supplementary Information file

Description of Additional Supplementary Files

Data set 1

Supplementary Movie

Reporting Summary

## Data Availability

Structural data supporting findings in this study have been deposited in the Protein Data Bank (PDB) and the Electron Microscopy Data Bank (EMDB). The accession codes of the cryo-EM maps and accompanying atomic models have been provided for: (1) OGA-L Catalytic Dimer (PDB ID: 9NE2, EMDB-49293), (2) A-chain of the OGA-L Catalytic Dimer (PDB ID: 9NE4, EMDB-49294), (3) B-chain of the OGA-L Catalytic Dimer (PDB ID: 9NE5, EMDB-49295), (4) OGA-L Dimer (EMDB-49296), (5) OGA-L Catalytic Dimer, A-chain extra density (EMDB-49297). Antibodies employed in this study can be found in Supplementary Table 2. *Additional data compared in this study from the protein databank: Alphafold2 Multimer, PDB ID: 5M7R, PDB ID: 5VVO, PDB ID:5UHK, PDB ID: 5M7S, PDBID:5UN9, PDB ID:5UHL, PDB ID: 5M7T, PDB ID: 5UHO, PDB ID: 8P0L, PDB ID: 9BA8, 9BA9, PDB ID: 1KX5*.
