# Supplementary material for: Human O-GlcNAcase catalytic-stalk dimer anchors flexible histone binding domains
Source: Commun Chem. 2025 Dec 9;9:8. doi: 10.1038/s42004-025-01813-7 (PMC12775002; doi:10.1038/s42004-025-01813-7)
Supplement: Supplementary file 1 — Supplementary Information file [file 42004_2025_1813_MOESM1_ESM.pdf]

# Human *O*-GlcNAcase catalytic-stalk dimer anchors flexible histone binding domains

Sarah B. Nyenhuis[1], Agata Steenackers[2,†], Mana Mohan Mukherjee[2], Jenny E. Hinshaw[1*] and John A. Hanover[2*]

[1]Laboratory of Molecular Biology, NIDDK, National Institutes of Health, Bethesda, MD, 20892, USA.
[2]Laboratory of Cell and Molecular Biology, NIDDK, National Institutes of Health, Bethesda, MD, 20892, USA.
[†]Present address: Versiti Blood Research Institute, Milwaukee, WI, 53226, USA

# Supplementary Information

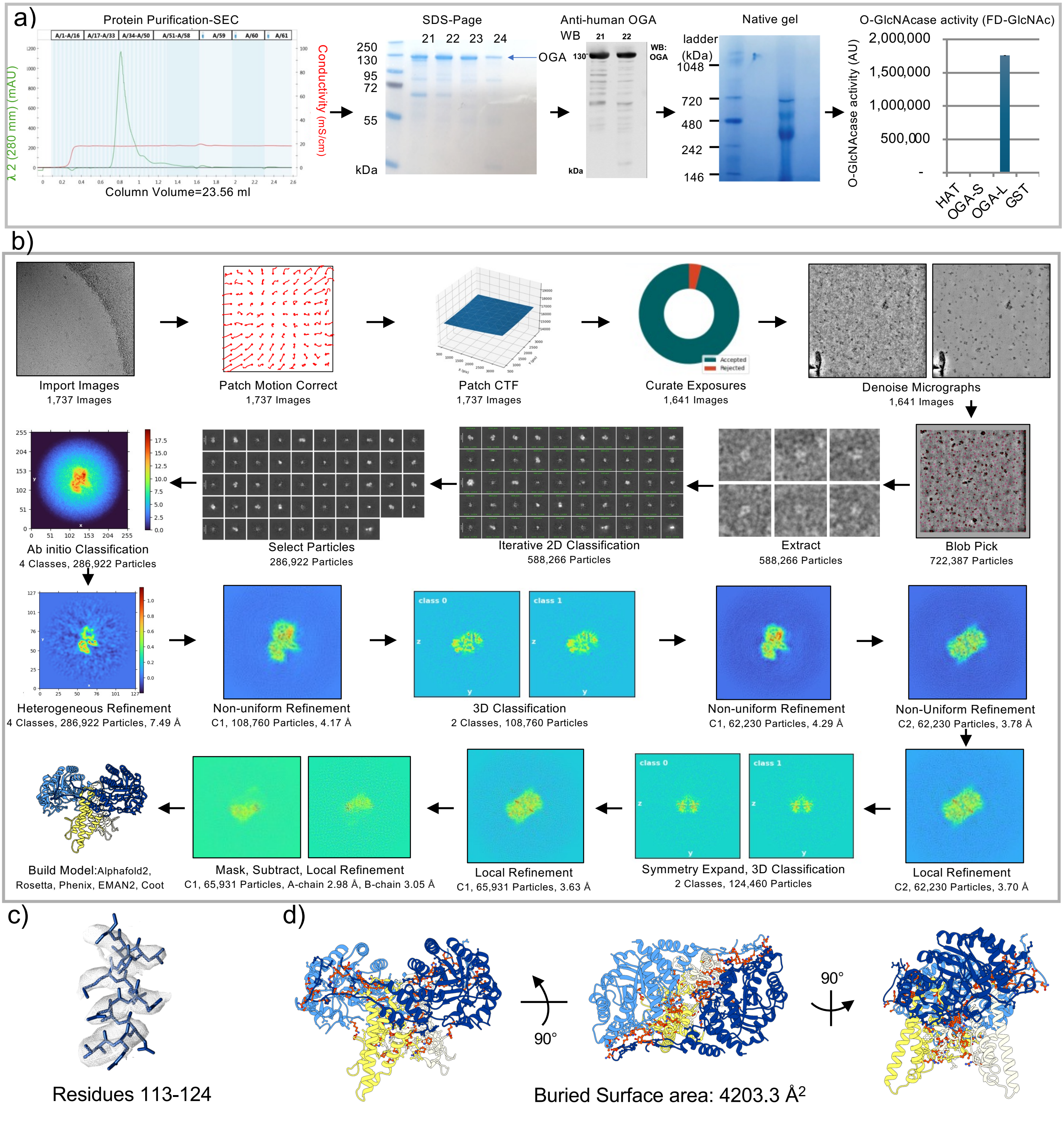


**Supplementary Figure 1: Purification, characterization, and workflow for determining the cryo-EM structure of the OGA-L dimer.** **(a)** Extracted protein was affinity purified using a His-column followed by size exclusion chromatography (left). Pooled fractions were analyzed by SDS-PAGE and western blotting using anti-human OGA. Migration of the purified protein on a native gel was consistent with dimerization of the protein with smaller amounts of higher oligomers. The activity of the purified OGA-L was determined using a fluorogenic substrate and compared to the expressed pHAT domain, the OGA-S isoform, and a His-tagged GST control (right). **(b)** An outline of cryo-EM data processing. Each dataset followed a standard workflow including patch motion correction, patch CTF correction, and exposure curation. Micrographs were denoised, then blob picker was used to select particles and subsequently extracted. Particles were iteratively 2D classified to remove junk and then aligned through *ab initio* reconstruction. The selected *ab initio* model was used for heterogeneous refinement, subsequent non-uniform refinement, and 3D classification using C1 symmetry. A single class was then non-uniformly refined again using C1 and then C2 symmetry for non-uniform and a local refinement. Once better aligned, particles were symmetry expanded and 3D classified again, then locally refined with C1 symmetry. Individual chains were then masked and subtracted to better resolve residues. The resulting maps were used to generate a model using Alphafold2 multimer, Rosetta v2021.16, Phenix v1.29.1-4487, Gaussian mixture model based atomic model refinement in EMAN2 v2.99.66, and Coot v0.9.8.92. The model of OGA is colored by domain, catalytic domain: dark blue, unstructured regions: gray, flexible helix: cyan; stalk: yellow. **(c)** A representative fit of the model to the map, highlighting residues 113-124 in the catalytic domain, map is shown with gray mesh. **(d)** OGA buried interfacial residues shown in three views (90° rotations) with the OGA model colored by domain: catalytic domain, dark blue; stalk, yellow highlighting the dimer interface. Interfacial residues in the dimer interface are shown as sticks with hydrogen bonding residues in orange. The buried surface area of the interface is 4203.3 Å, determined using PDBePISA.

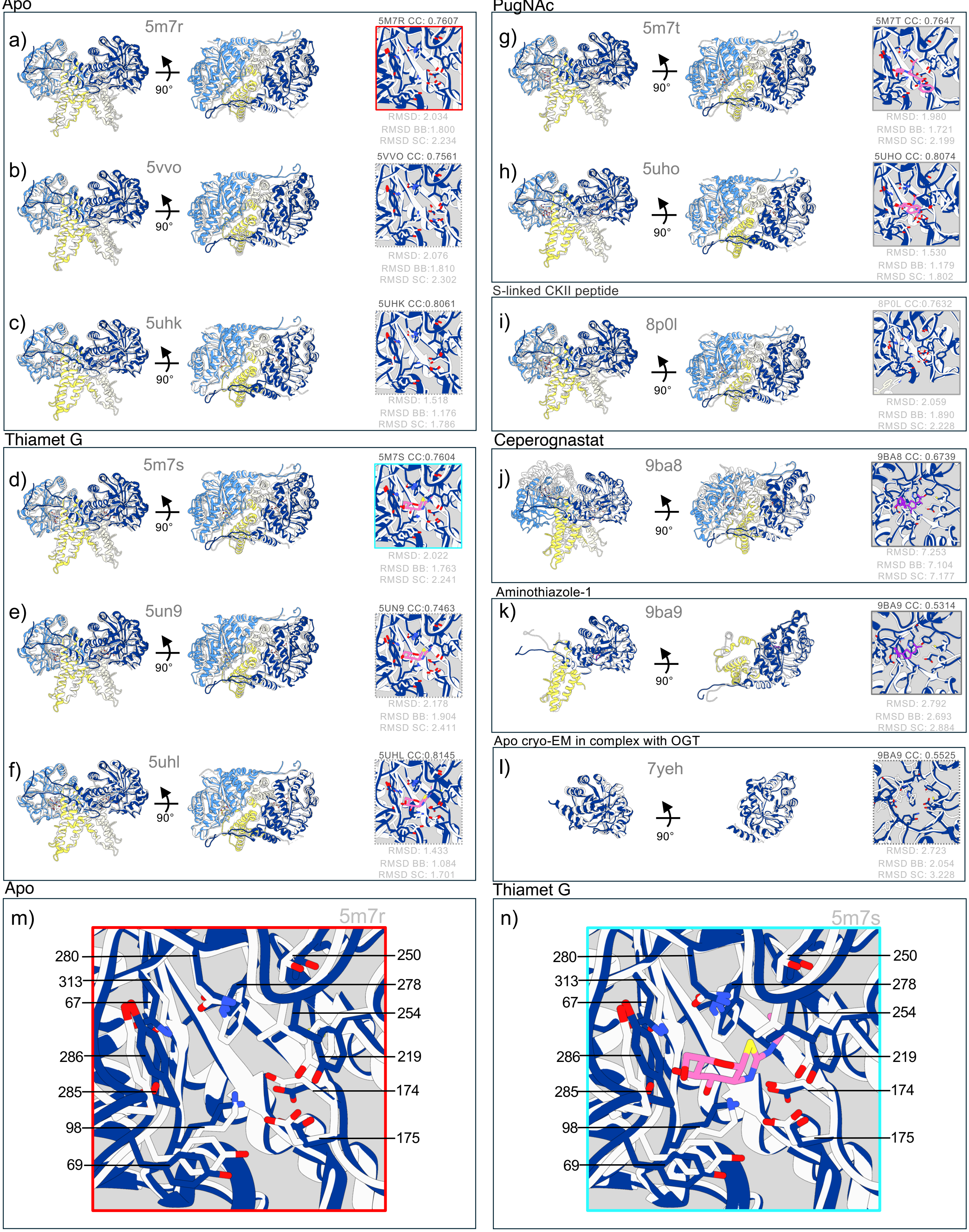


**Supplementary Figure 2: OGA model compared to crystal structures. (a-k)** Cryo-EM OGA model colored by domain (catalytic domain, dark blue; stalk, yellow) overlaid with crystal structures (gray). Right, magnified view of the active site with or without substrate bound with cross-correlation and RMSD values between the models shown above and below respectively. RMSD values correspond to calculations including all atoms (RMSD), backbone atoms (RMSD BB), and sidechain atoms (RMSD SC) as annotated in Supplementary Table 2. (**a-c**) Apo crystal structures, 5m7r, 5vvo, and 5uhk. (**d-f** )Thiamet G (pink) bound crystal structures 5m7s, 5un9, and 5uhl. (**g-h**) PugNAc (pink) bound crystal structures 5m7t and 5uho. (**i**) S-linked CDKII peptide bound crystal structure 8p0l. (**j**) Ceperognastat (purple) bound crystal structure 9ba8. (**k**) Aminothiazole-1 (purple) bound crystal structure 9ba9. **i** OGA model colored as in (**a**) overlaid with the Apo cryo-EM of OGA in complex with OGT, 7yeh (gray). Right panel, magnified view of the active site, displaying residues involved in the ThiametG binding (dashed gray box) with cross-correlation and RMSD values between the models shown above and below. **(m,n)** Zoom of OGA model colored blue overlaid with the Apo crystal structure 5m7r (**a**, red box) and the Thiamet G bound crystal structure 5m7s (**d**, blue box) colored white highlighting the active site, with residues involved in Thiamet G binding shown as sticks.

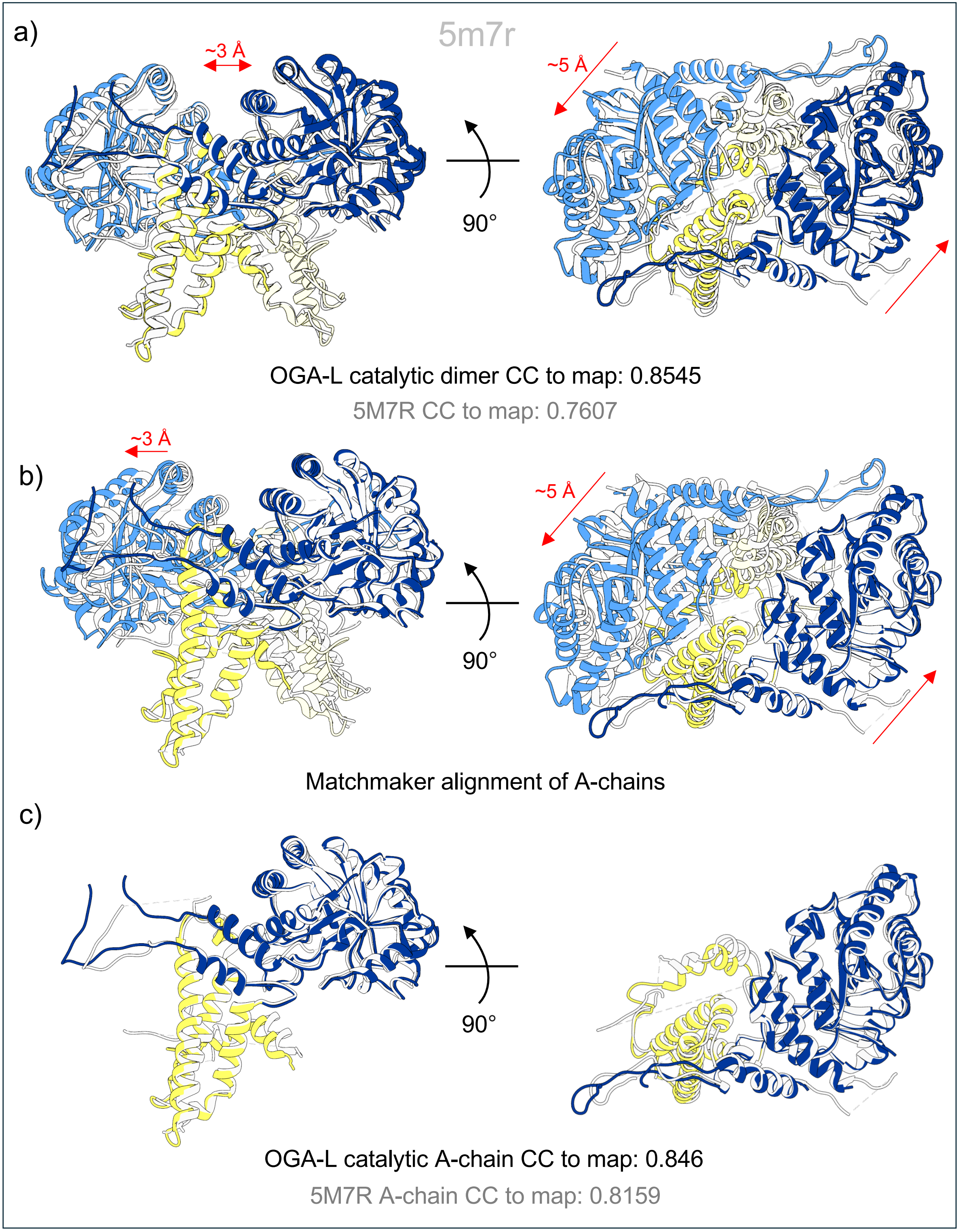


**Supplementary Figure 3: OGA model compared to the 5m7r, apo crystal structures. (a)** An overlay of the cryo-EM OGA model colored by domain (catalytic domain, dark blue; stalk, yellow) and the 5m7r crystal structure (gray), resulting from fitting models into the cryo-EM dimer map. A 3 Å opening between the monomers and a 5 Å translational shift is observed between 5m7r and our cryo-EM model (red arrows). Below, cross-correlations (CC) of the models fit into the cryo-EM map using Chimera. (**b)** An overlay of the cryo-EM OGA model colored as in (**a**) and the 5m7r crystal structure (gray), the overlay is a product of aligning the A-chains using matchmaker. (**c)** An overlay of the A-chain of the cryo-EM OGA model colored as in (**a**) and the 5m7r crystal structure A-chain (gray), the overlay is a product of aligning the A-chains using matchmaker. Below, cross-correlations (CC) of the models fit into the cryo-EM map using Chimera.

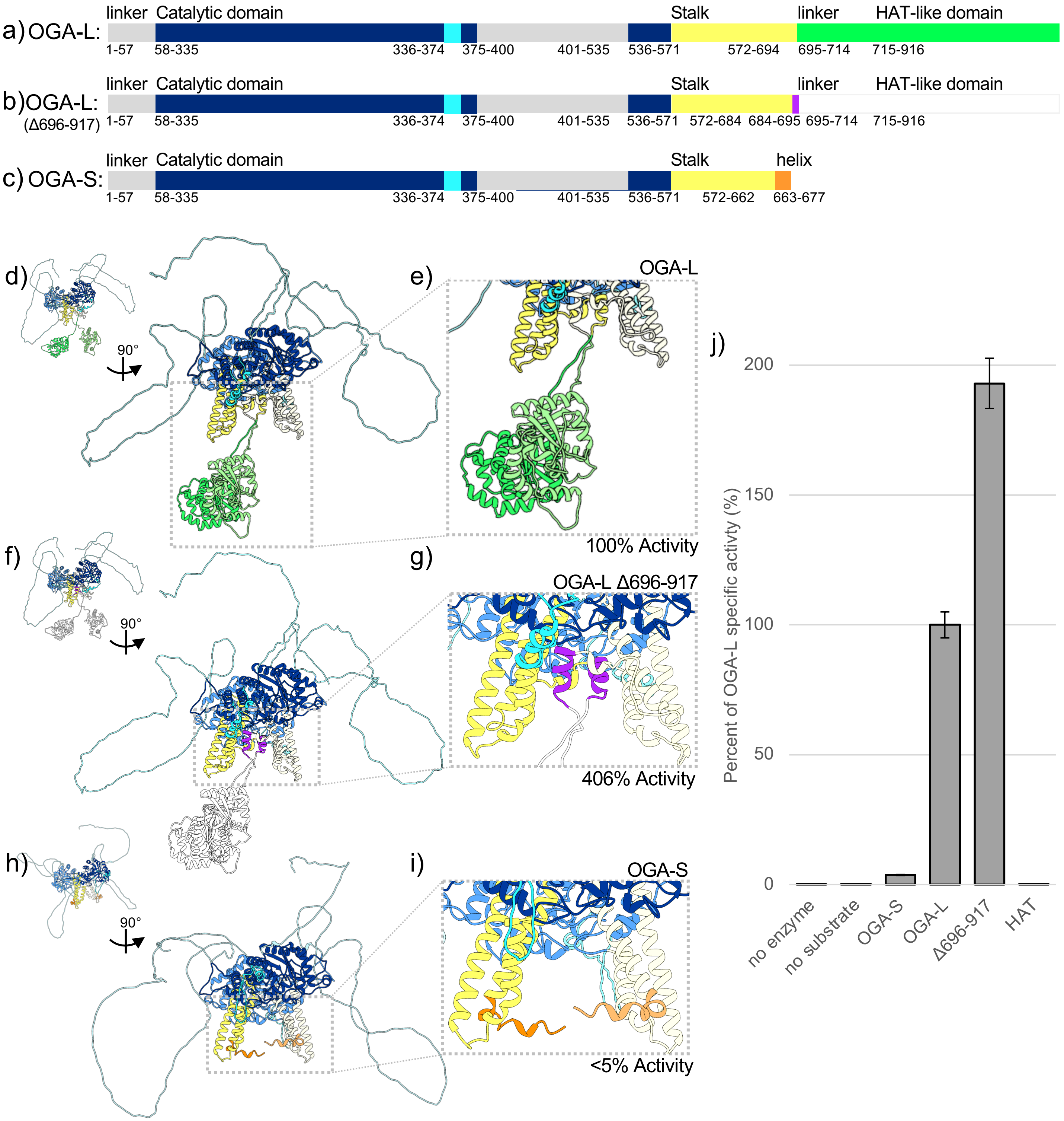


**Supplementary Figure 4: A comparison between OGA-L and OGA-S. (a,b,c)** Sequence diagram of OGA-L, OGA-L (Δ696-917), and OGA-S colored by domain, highlighting flexible regions. OGA catalytic domain: dark blue, unstructured regions: gray, flexible helix,: cyan; stalk: yellow; linker and pHAT domain: green; trans helix: purple; deleted regions: white. (**d**) A bar-chart of the catalytic activity of the different constructs. Assays were conducted in triplicate, and the mean and standard error of the mean (error bars) are indicated. **(e)** OGA-L model colored as in (**a**) and a 90° rotation of the model. Right**,** Magnified image (dashed gray box) highlighting the linker, the pHAT domain, and the catalytic activity. **(f)** OGA-L (Δ696-917) model colored as in (**b**) and a 90° rotation of the model. (**g**) Magnified image (dashed gray box) highlighting the linker, the trans helix present in OGA-L, and the catalytic activity. (**h**) An Alphafold2 model of OGA-S colored as in (**c**) and a 90° rotation of the model. **(i)** Magnified image (dashed gray box) highlighting the unique a-helical extension and the catalytic activity. (**j**) Specific catalytic activity of OGA-S, OGA-L, Δ696-919, and HAT domain expressed as a percentage (%) of OGA-L specific activity (N = 6 individual experimental replicates)

```
sp|OGA-S      MVQKESQATLEERESELSSNPAASAGASLEPPAAPAPGEDNPAGAGGAAVAGAAGGARRF   60
sp|OGA-3      MVQKESQATLEERESELSSNPAASAGASLEPPAAPAPGEDNPAGAGGAAVAGAAGGARRF   60
sp|OGA-L      MVQKESQATLEERESELSSNPAASAGASLEPPAAPAPGEDNPAGAGGAAVAGAAGGARRF   60
sp|OGA-4      MVQKESQATLEERESELSSNPAASAGASLEPPAAPAPGEDNPAGAGGAAVAGAAGGARRF   60
              ************************************************************

sp|OGA-S      LCGVVEGFYGRPWVMEQRKELFRRLQKWELNTYLYAPKDDYKHRMFWREMYSVEEAEQLM   120
sp|OGA-3      LCGVVEGFYGRPWVMEQRKELFRRLQKWELNTYLYAPKDDYKHRMFWREMYSVEEAEQLM   120
sp|OGA-L      LCGVVEGFYGRPWVMEQRKELFRRLQKWELNTYLYAPKDDYKHRMFWREMYSVEEAEQLM   120
sp|OGA-4      LCGVVEGFYGRPWVMEQRKELFRRLQKWELNTYLYAPKDDYKHRMFWREMYSVEEAEQLM   120
              ************************************************************

sp|OGA-S      TLISAAREYEIEFIYAISPGLDITFSNPKEVSTLKRKLDQVSQFGCRSFALLFDDIDHNM   180
sp|OGA-3      TLISAAREYEIEFIYAISPGLDITFSNPKEVSTLKRKLDQVSQFGCRSFALLFDDIDHNM   180
sp|OGA-L      TLISAAREYEIEFIYAISPGLDITFSNPKEVSTLKRKLDQVSQFGCRSFALLFDDIDHNM   180
sp|OGA-4      TLISAAREYEIEFIYAISPGLDITFSNPKEVSTLKRKLDQVSQFGCRSFALLFDDIDHNM   180
              ************************************************************

sp|OGA-S      CAADKEVFSSFAHAQVSITNEIYQYLGEPETFLFCPTEYCGTFCYPNVSQSPYLRTVGEK   240
sp|OGA-3      CAADKEVFSSFAHAQVSITNEIYQYLGEPETFLFCPTEYCGTFCYPNVSQSPYLRTVGEK   240
sp|OGA-L      CAADKEVFSSFAHAQVSITNEIYQYLGEPETFLFCPTEYCGTFCYPNVSQSPYLRTVGEK   240
sp|OGA-4      CAADKEVFSSFAHAQVSITNEIYQYLGEPETFLFCPTEYCGTFCYPNVSQSPYLRTVGEK   240
              ************************************************************

sp|OGA-S      LLPGIEVLWTGPKVVSKEIPVESIEEVSKIIKRAPVIWDNIHANDYDQKRLFLGPYKGRS   300
sp|OGA-3      LLPGIEVLWTGPKVVSKEIPVESIEEVSKIIKRAPVIWDNIHANDYDQKRLFLGPYKGRS   300
sp|OGA-L      LLPGIEVLWTGPKVVSKEIPVESIEEVSKIIKRAPVIWDNIHANDYDQKRLFLGPYKGRS   300
sp|OGA-4      LLPGIEVLWTGPKVVSKEIPVESIEEVSKIIKRAPVIWDNIHANDYDQKRLFLGPYKGRS   300
              ************************************************************

sp|OGA-S      TELIPRLKGVLTNPNCEFEANYVAIHTLATWYKSNMNGVRKDVVMTDSEDSTVSIQIKLE   360
sp|OGA-3      TELIPRLKGVLTNPNCEFEANYVAIHTLATWYKSNMNGVRKDVVM---------------   345
sp|OGA-L      TELIPRLKGVLTNPNCEFEANYVAIHTLATWYKSNMNGVRKDVVMTDSEDSTVSIQIKLE   360
sp|OGA-4      TELIPRLKGVLTNPNCEFEANYVAIHTLATWYKSNMNGVRKDVVM---------------   345
              *********************************************

sp|OGA-S      NEGSDEDIETDVLYSPQMALKLALTEWLQEFGVPHQYSSRQVAHSGAKASVVDGTPLVAA   420
sp|OGA-3      -------------------------------------SRQVAHSGAKASVVDGTPLVAA   367
sp|OGA-L      NEGSDEDIETDVLYSPQMALKLALTEWLQEFGVPHQYSSRQVAHSGAKASVVDGTPLVAA   420
sp|OGA-4      -------------------------------------SRQVAHSGAKASVVDGTPLVAA   367
                                                    **********************

sp|OGA-S      PSLNATTVVTTVYQEPIMSQGAALSGEPTTLTKEEEKKQPDEEPMDMVVEKQEETDHKND   480
sp|OGA-3      PSLNATTVVTTVYQEPIMSQGAALSGEPTTLTKEEEKKQPDEEPMDMVVEKQEETDHKND   427
sp|OGA-L      PSLNATTVVTTVYQEPIMSQGAALSGEPTTLTKEEEKKQPDEEPMDMVVEKQEETDHKND   480
sp|OGA-4      PSLNATTVVTTVYQEPIMSQGAALSGEPTTLTKEEEKKQPDEEPMDMVVEKQEETDHKND   427
              ************************************************************

sp|OGA-S      NQILSEIVEAKMAEELKPMDTDKESIAESKSPEMSMQEDCISDIAPMQTDEQTNKEQFVP   540
sp|OGA-3      NQILSEIVEAKMAEELKPMDTDKESIAESKSPEMSMQEDCISDIAPMQTDEQTNKEQFVP   487
sp|OGA-L      NQILSEIVEAKMAEELKPMDTDKESIAESKSPEMSMQEDCISDIAPMQTDEQTNKEQFVP   540
sp|OGA-4      NQILSEIVEAKMAEELKPMDTDKESIAESKSPEMSMQEDCISDIAPMQTDEQTNKEQFVP   487
              ************************************************************

sp|OGA-S      GPNEKPLYTAEPVTLEDLQLLADLFYLPYEHGPKGAQMLREFQWLRANSSVVSVNCKGKD   600
sp|OGA-3      GPNEKPLYTAEPVTLEDLQLLADLFYLPYEHGPKGAQMLREFQWLRANSSVVSVNCKGKD   547
sp|OGA-L      GPNEKPLYTAEPVTLEDLQLLADLFYLPYEHGPKGAQMLREFQWLRANSSVVSVNCKGKD   600
sp|OGA-4      GPNEKPLYTAEPVTLEDLQLLADLFYLPYEHGPKGAQMLREFQWLRANSSVVSVNCKGKD   547
              ************************************************************

sp|OGA-S      SEKIEEWRSRAAKFEEMCGLVMGMFTRLSNCANRTILYDMYSYVWDIKSIMSMVKSFVQW   660
sp|OGA-3      SEKIEEWRSRAAKFEEMCGLVMGMFTRLSNCANRTILYDMYSYVWDIKSIMSMVKSFVQW   607
sp|OGA-L      SEKIEEWRSRAAKFEEMCGLVMGMFTRLSNCANRTILYDMYSYVWDIKSIMSMVKSFVQW   660
sp|OGA-4      SEKIEEWRSRAAKFEEMCGLVMGMFTRLSNCANRTILYDMYSYVWDIKSIMSMVKSFVQW   607
              ************************************************************

sp|OGA-S      LGRCTRNNLFS--SNILSL-----------------------------------------   677
sp|OGA-3      LGCRSHSSAQFLIGDQEPWAFRGGLAGEF--------------QPPPLTPTSKVYTIRPY   653
sp|OGA-L      LGCRSHSSAQFLIGDQEPWAFRGGLAGEFQRLLPIDGANDLFFQPPPLTPTSKVYTIRPY   720
sp|OGA-4      LGCRSHSSAQFLIGDQEPWAFRGGLAGEFQRLLPIDGANDLFFQPPPLTPTSKVYTIRPY   667
              **  ::..     .:

sp|OGA-S      ------------------------------------------------------------   677
sp|OGA-3      FPKDEASVYKICREMYDDGVGLPFQSQPDLIGDKLVGGLLSLSLDYCFVLEDEDGICGYA   713
sp|OGA-L      FPKDEASVYKICREMYDDGVGLPFQSQPDLIGDKLVGGLLSLSLDYCFVLEDEDGICGYA   780
sp|OGA-4      FPKDEASVYKICREMYDDGVGLPFQSQPDLIGDKLVGGLLSLSLDYCFVLEDEDGICGYA   727

sp|OGA-S      ------------------------------------------------------------   677
sp|OGA-3      LGTVDVTPFIKKCKISWIPFMQEKYTKPNGDKELSEAEKIMLSFHEEQEVLPETFLANFP   773
sp|OGA-L      LGTVDVTPFIKKCKISWIPFMQEKYTKPNGDKELSEAEKIMLSFHEEQEVLPETFLANFP   840
sp|OGA-4      LGTVDVTPFIKKCKISWIPFMQEKYTKPNGDKELSEAEKIMLSFHEEQEVLPETFLANFP   787

sp|OGA-S      ------------------------------------------------------------   677
sp|OGA-3      SLIKMDIHKKVTDPSVAKSMMACLLSSLKANGSRGAFCEVRPDDKRILEFYSKLGCFEIA   833
sp|OGA-L      SLIKMDIHKKVTDPSVAKSMMACLLSSLKANGSRGAFCEVRPDDKRILEFYSKLGCFEIA   900
sp|OGA-4      SLIKMDIHKKVTDPSVAKSMMACLLSSLKANGSRGAFCEVRPDDKRILEFYSKLGCFEIA   847

sp|OGA-S      ---------------- 677
sp|OGA-3      KMEGFPKDVVILGRSL 849
sp|OGA-L      KMEGFPKDVVILGRSL 916
sp|OGA-4      KMEGFPKDVVILGRSL 863
```

**Supplementary Figure 5: Sequence alignment of OGA isoforms.** The four isoforms of OGA are colored by domain. OGA catalytic domain: dark blue, unstructured regions: grey, flexible helix: cyan; stalk: yellow; trans helix present in OGA-L: purple; linker and pHAT domain: green. The short isoform, OGA-S, lacks the pHAT domain and includes a unique a-helix colored orange.

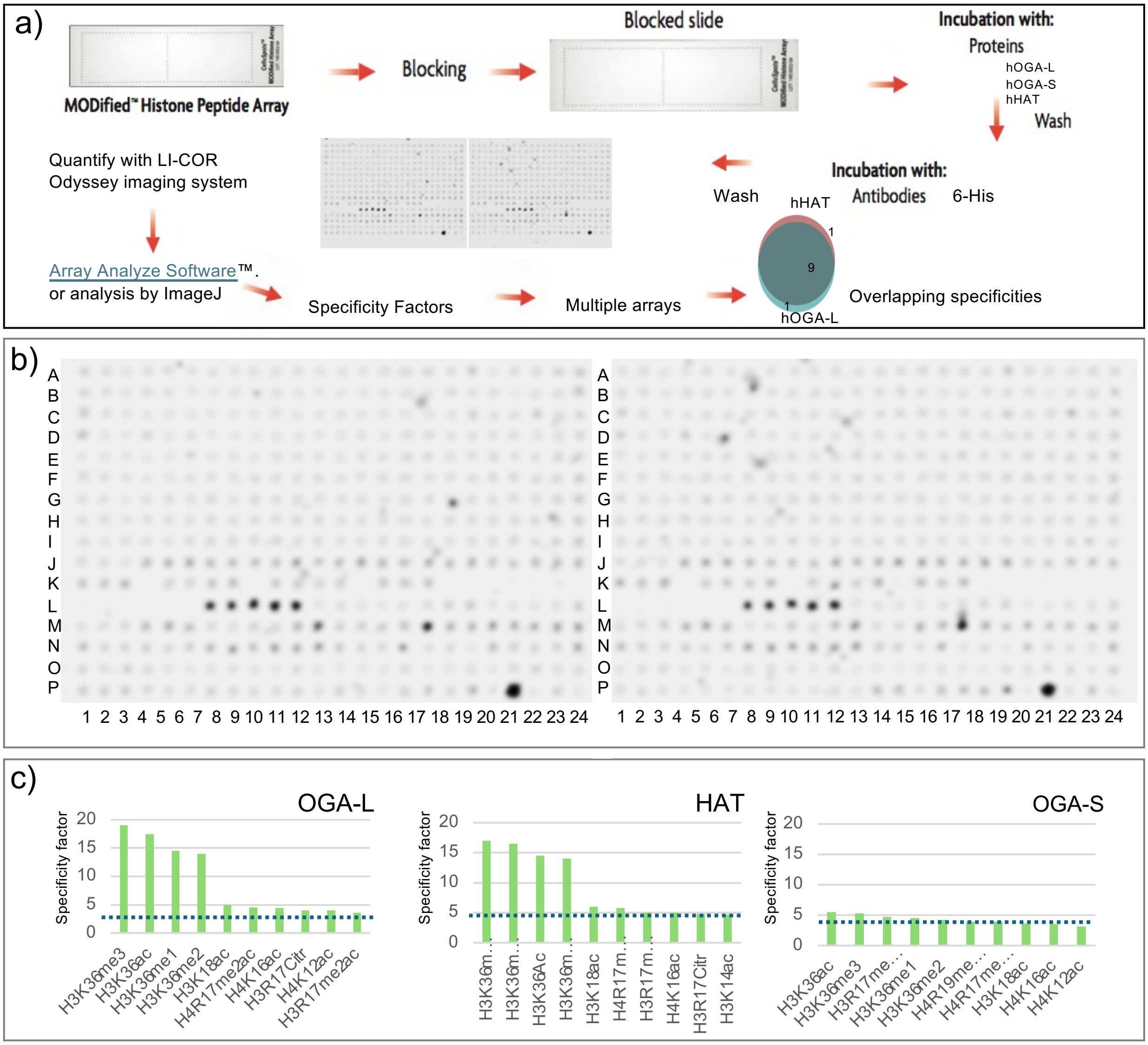


d) Average Specificity Factors:

| | H3K36Me3 | H3K36ac | H3K36me1 | H3K36me2 | H3K18ac | H4R17me2ac |
|---|---|---|---|---|---|---|
| OGA-S | 5.3 | 5.5 | 4.2 | 4 | 3.8 | 3.8 |
| HAT | 17 | 15 | 17 | 14 | 6 | 5.5 |
| OGA-L | 19 | 18 | 14 | 13 | 5 | 4.5 |

**Supplementary Figure 6. Workflow for Probing Modified™ Histone Arrays with OGA-L and OGA Subdomains.** (**a**) The OGA-L and each of the subdomains were expressed as fusion proteins with His-tag and C-term tag epitopes. Proteins were purified by affinity chromatography and after a blocking step were incubated with Histone arrays as described in Materials and Methods. After washing, arrays were probed with either anti-His Tag or C-term antibodies as described in materials and methods. After extensive washing, binding was quantified using a LI-COR Odyssey imaging system and analyzed using the Array Analyze Software to create a specificity index. Three of these duplicate arrays were analyzed and peptides showing specificity factors greater than 5 in all three probed duplicate arrays were judged to be specific binding interactions. Those interactions shared by the pHAT and OGA-L domains were (in order of specificity factor): $H3K36^{Me3}$>$H3K36^{Me2}$>$H3K36^{Ac}$>$H3K36^{Me1}$>$H3K36^{unmodifed}$>H4 1-19 $K^{5,8,12,16Ac}$> H4K16Ac>$H3R17^{Me}$2>$H3R17^{Citr}$>$H3K18^{Ac}$>$H4R17^{Me2}$. (**b**) Representative blots with numbering corresponding to the MODified_Histone_Peptide_Array Grid shown in Supplementary Table 3. (**c**) Arrays were quantified using the Array Analyze Software as described by the manufacturer to produce the "Specificity Factor" plots for the arrays for OGA-L, pHAT domain and OGA-S. These graphs consider the background and positive control (See P21) and show only singly modified histone peptides and exclude unmodified histones. **(d)** Specificity factors for the most significant positive modified peptides observed.

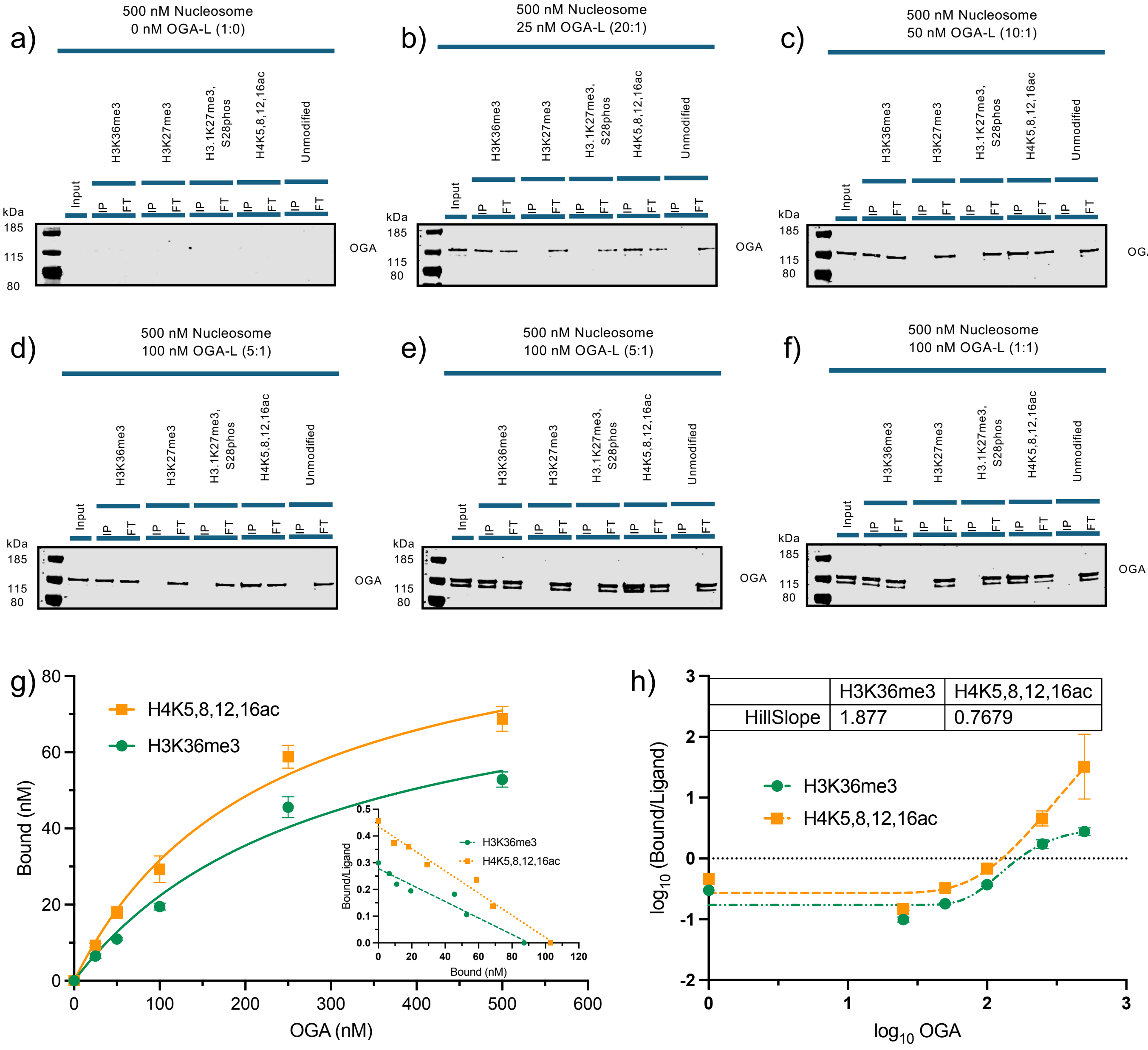


**Supplementary Figure 7. Blots and Quantification of recombinant Nucleosome binding data. (a-f)** Biotinylated recombinant nucleosomes (500 nM) were incubated at 4 °C with the indicated concentrations of OGA-L. The binding partners were harvested with streptavidin magnetic beads and washed prior to western blotting and detection using OGA-L-specific antibodies. (**g, h**) Quantification was performed by IR imaging as described in "Experimental Procedures". Resultant values for binding are shown as a saturation or Scatchard plot **(g)** and analyzed for as Hill Slop **(h)**. Error bars representing the standard deviation centered on the mean. N = 3 independent experimental replicates.

# A) Unprocessed blot for Supplementary Figure 4a to 4f

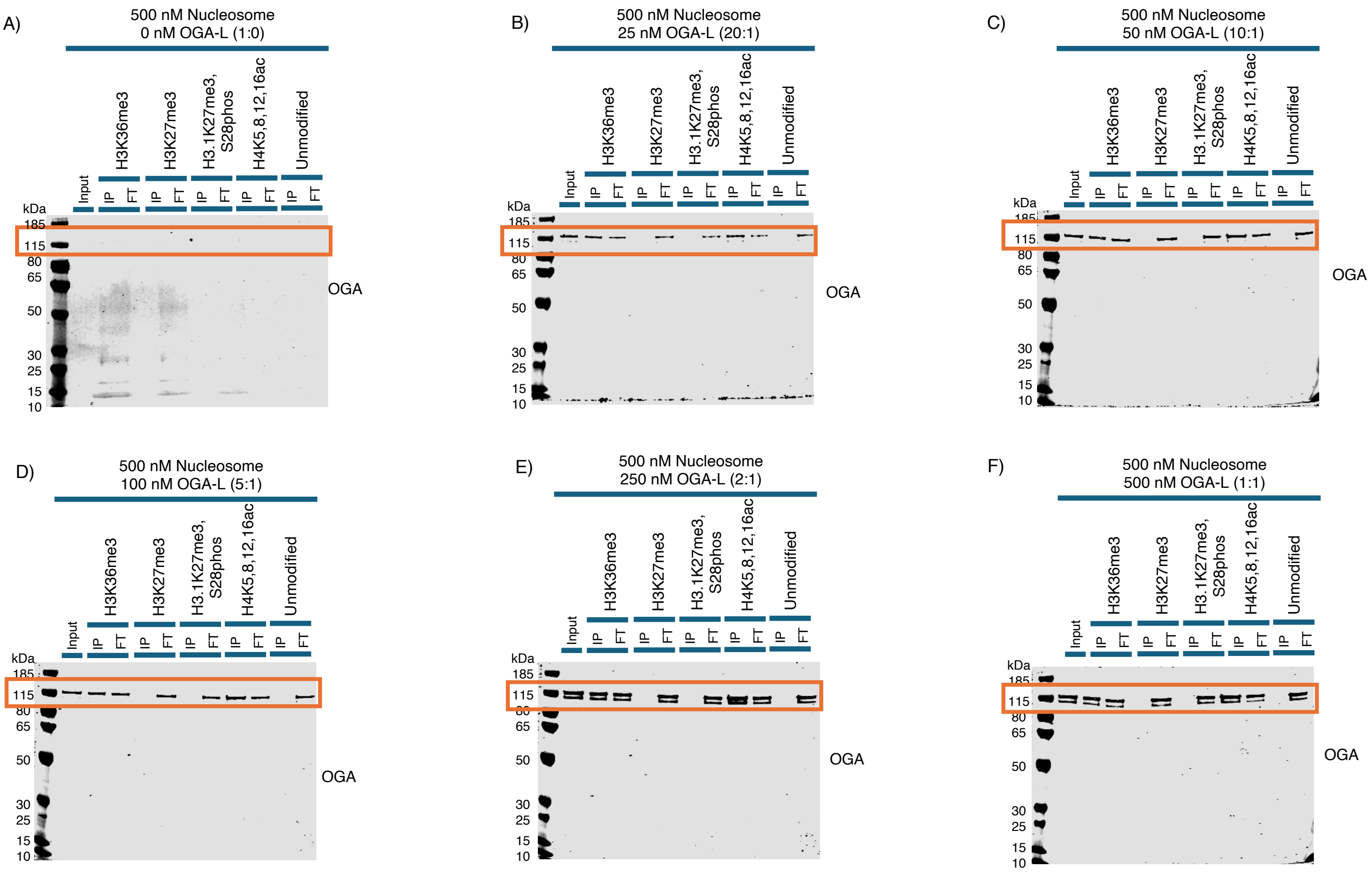


**Supplementary Figure 8.** Full uncropped western blots showing *OGA-L* proteins in histone bound and flow through fractions when binding assay of 500 nM Nucleosomes were performed with (A) 0 nM OGA-L; (B) 25 nM OGA-L (C) 50 nM OGA-L; (D) 100 nM OGA-L; (E) 250 nM OGA-L; and (F) 500 nM OGA-L

A) Unprocessed blot for Figure 4c B) Unprocessed blot for Figure 4d

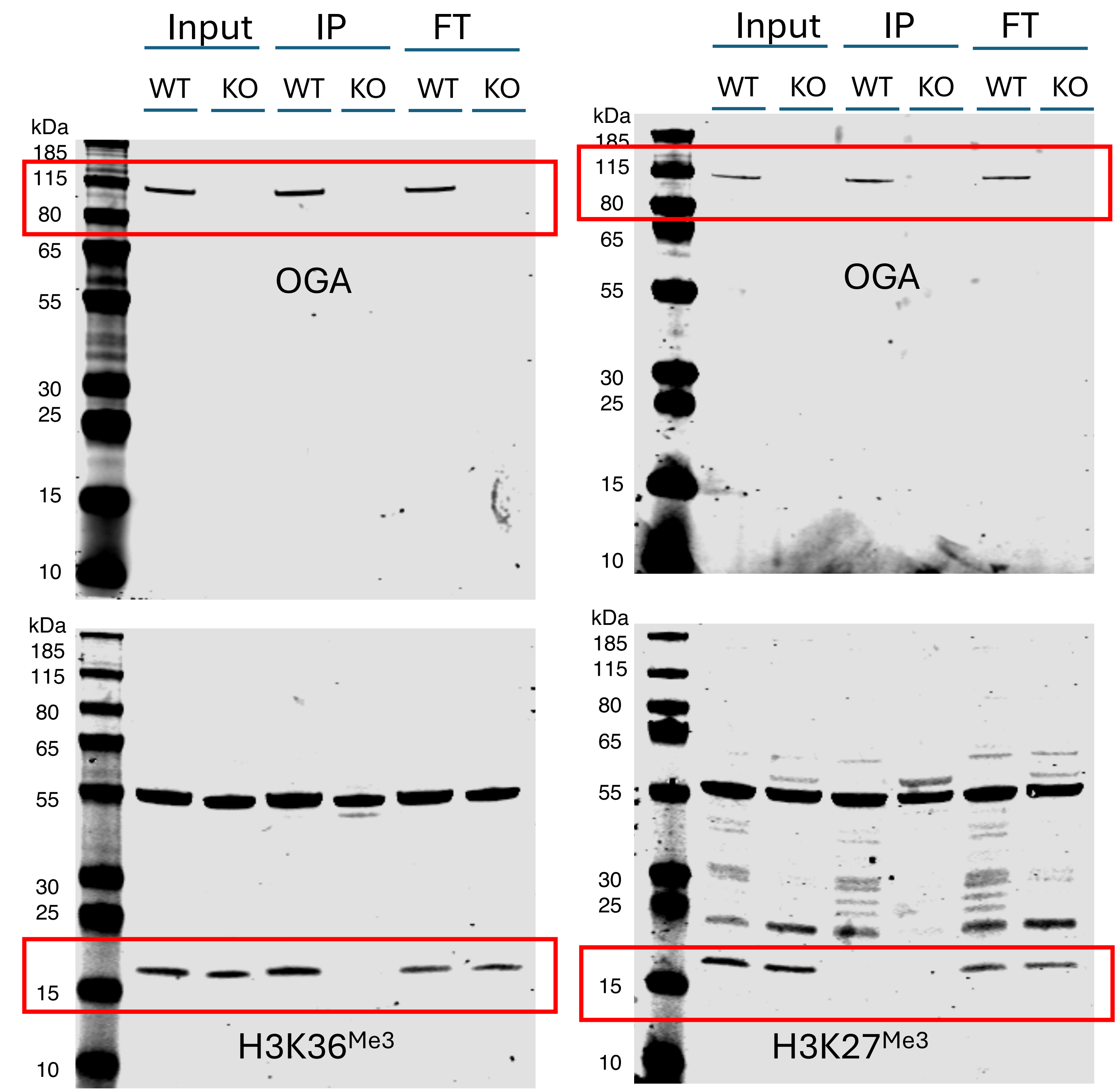


**Supplementary Figure 9.** Full uncropped western blots showing *OGA-L,* and histone proteins bands in immunoprecipitation experiment with OGA WT and OGA KO MEF cells for (A) H3K36$^{Me3}$ and (B) H3K27$^{Me3}$

| OGA-L (human) | | | | | |
|---|---|---|---|---|---|
| | Average Buried Surface Area (Å2) | Residues which H-bond | Residues which form salt bridges | Buried Interface Residues (A-chain) | Buried Interface Residues (B-chain) |
| **Catalytic dimer interface** | 4203.3 | C631 (A):Y101 (B), S629 (A):Y101 (B), E544 (A):N147 (B), N543 (A):A182 (B), N543 (A):A183 (B), Y641 (A):Q288 (B), D646 (A):K289 (B), E109 (A):H395 (B), D142 (A):Y397 (B), M105 (A):Y397 (B), E109 (A):S398 (B), T144(A):K545 (B), D142 (A):Y548 (B), E688 (A):H571 (B), L692 (A):R586 (B), D99 (A):R634 (B), D99 (A):Y638 (B), G70 (A):Y638 (B), P694 (A):W679 (B), Q690 (A):R682 (B), D287 (A):R682 (B), W679 (A):Q690 (B), Y101 (A):S629 (B), Y101 (A):C631 (B), R108 (A):Y397 (B), N147 (A):E544 (B), A182 (A):N543 (B), A183 (A):N543 (B), K289 (A):D646 (B), H395 (A):E109 (B), S398 (A):E109 (B), K545 (A):T144 (B), Y548 (A):D142 (B), Y548 (A):M105 (B), R586 (A):L692 (B), Y638 (A):G70 (B), W679 (A):P694 (B) | D675 (A):K253 (B), D646 (A):K289 (B), E109 (A):H395 (B), E688 (A):H571 (B), D287 (A):R682 (B), K253 (A):D675 (B), K289 (A):D646 (B), H395 (A):E109 (B) | Y69, G70, R71, P72, D99, D100, Y101, K102, M105, F106, W107, R108, E109, G140, L141, D142, I143, T144, N147, K149, E150, T153, D177, N179, M180, C181, A182, A183, T222, K253, D285, Y286, D287, Q288, K289, R290, H395, Q396, Y397, S398, E536, F538, N543, E544, K545, P546, L547, Y548, A550, D563, L564, F565, P568, Y569, E570, H571, G575, A576, M578, L579, F582, Q583, R586, F614, S629, N630, C631, A632, R634, Y638, D639, Y641, S642, Y643, W645, D646, I647, S649, I650, M651, M653, V654, F657, W660, Q670, F671, D675, Q676, E677, P678, W679, A680, F681, R682, G683, G684, L685, A686, E688, F689, Q690, R691, L692, L693, P694, I695, D696 | Y69, G70, R71, P72, D99, D100, Y101, K102, M105, F106, R108, E109, G140, L141, D142, I143, T144, F145, N147, K149, E150, T153, D177, N179, M180, C181, A182, A183, T222, K253, D285, Y286, D287, Q288, K289, R290, C316, H395, Q396, Y397, S398, E536, Q537, F538, N543, E544, K545, P546, L547, Y548, A550, D563, L564, P568, Y569, E570, H571, G575, A576, M578, L579, F582, Q583, R586, F614, S629, N630, C631, A632, R634, Y638, D639, Y641, S642, Y643, W645, D646, I647, S649, I650, M651, M653, V654, F657, F671, D675, Q676, E677, W679, A680, F681, R682, G683, G684, L685, A686, G687, E688, F689, Q690, R691, L692, L693, P694, I695, D696 |

**Supplementary Table 1: OGA-L dimer interface**. Buried surface area and residues for each interface as identified by the server PDBePISA.

| | RMSD all atoms | RMSD Backbone | RMSD Sidechains | RMSD Cα |
|---|---|---|---|---|
| **Apo** | | | | |
| **5m7r** | 2.034 (6592 atoms) | 1.800 (3216 atoms) | 2.234 (3376 atoms) | 1.795 (804 atoms) |
| **5vvo** | 2.076 (6576 atoms) | 1.810 (3216 atoms) | 2.302 (3360 atoms) | 1.791 (804 atoms) |
| **5uhk** | 1.518 (6552 atoms) | 1.176 (3208 atoms) | 1.786 (3344 atoms) | 1.158 (802 atoms) |
| **ThiametG** | | | | |
| **5m7s** | 2.022 (6592 atoms) | 1.763 (3216 atoms) | 2.241 (3376 atoms) | 1.757 (804 atoms) |
| **5un9** | 2.178 (6571 atoms) | 1.904 (3212 atoms) | 2.411 (3359 atoms) | 1.894 (803 atoms) |
| **5uhl** | 1.433 (6552 atoms) | 1.084 (3208 atoms) | 1.701 (3344 atoms) | 1.060 (802 atoms) |
| **PugNAc** | | | | |
| **5m7t** | 1.980 (6592 atoms) | 1.721 (3216 atoms) | 2.199 (3376 atoms) | 1.715 (804 atoms) |
| **5uho** | 1.530 (6552 atoms) | 1.179 (3208 atoms) | 1.802 (3344 atoms) | 1.154 (802 atoms) |
| **S-linked CKII peptide** | | | | |
| **8p0l** | 2.059 (6275 atoms) | 1.890 (3216 atoms) | 2.228 (3043 atoms) | 1.883 (804 atoms) |
| **Ceperognastat** | | | | |
| **9ba8** | 7.253 (6495 atoms) | 7.104 (3216 atoms) | 7.177 (3287 atoms) | 7.068 (804 atoms) |
| **Aminothiazole-1** | | | | |
| **9ba9** | 2.792 (3230 atoms) | 2.693 (1608 atoms) | 2.884 (1622 atoms) | 2.644 (402 atoms) |
| **Apo cryo-EM in complex with OGT** | | | | |
| **7yeh** | 2.723 (2409 atoms) | 2.054 (1172 atoms) | 3.228 (1237 atoms) | 2.057 (293 atoms) |

**Supplementary Table 2: RMSD values of OGA-L catalytic dimer and published crystal structures**. RMSD values were calculated in Pymol, for all atoms, backbone atoms, sidechains, and Ca atoms.

# Active Motif
# MODified™ Histone Peptide Array*
# Catalog Nos. 13001 & 13005

| *Modification number* | *Peptide location* | *Peptide sequence* | *to array* | *name* | *Mod1* | *Mod2* | *Mod 3* | *Mod 4* | *N-terminus* |
|---|---|---|---|---|---|---|---|---|---|
| 1 | A 1 | A R T K Q T A R K S T G G K A P R K Q | ▶ | H3 1-19 | unmod | | | | free |
| 2 | A 2 | A Rme2s T K Q T A R K S T G G K A P R K Q | ▶ | H3 1-19 | R2me2s | | | | free |
| 3 | A 3 | A Rme2a T K Q T A R K S T G G K A P R K Q | ▶ | H3 1-19 | R2me2a | | | | free |
| 4 | A 4 | A Cit T K Q T A R K S T G G K A P R K Q | ▶ | H3 1-19 | R2Citr | | | | free |
| 5 | A 5 | A R pT K Q T A R K S T G G K A P R K Q | ▶ | H3 1-19 | T3P | | | | free |
| 6 | A 6 | A R T Kme1 Q T A R K S T G G K A P R K Q | ▶ | H3 1-19 | K4me1 | | | | free |
| 7 | A 7 | A R T Kme2 Q T A R K S T G G K A P R K Q | ▶ | H3 1-19 | K4me2 | | | | free |
| 8 | A 8 | A R T Kme3 Q T A R K S T G G K A P R K Q | ▶ | H3 1-19 | K4me3 | | | | free |
| 9 | A 9 | A R T Kac Q T A R K S T G G K A P R K Q | ▶ | H3 1-19 | K4ac | | | | free |
| 10 | A10 | A R T K Q T A Rme2s K S T G G K A P R K Q | ▶ | H3 1-19 | R8me2s | | | | free |
| 11 | A11 | A R T K Q T A Rme2a K S T G G K A P R K Q | ▶ | H3 1-19 | R8me2a | | | | free |
| 12 | A12 | A R T K Q T A Cit K S T G G K A P R K Q | ▶ | H3 1-19 | R8Citr | | | | free |
| 13 | A13 | A R T K Q T A R Kme1 S T G G K A P R K Q | ▶ | H3 1-19 | K9me1 | | | | free |
| 14 | A14 | A R T K Q T A R Kme2 S T G G K A P R K Q | ▶ | H3 1-19 | K9me2 | | | | free |
| 15 | A15 | A R T K Q T A R Kme3 S T G G K A P R K Q | ▶ | H3 1-19 | K9me3 | | | | free |
| 16 | A16 | A R T K Q T A R Kac S T G G K A P R K Q | ▶ | H3 1-19 | K9ac | | | | free |
| 17 | A17 | A R T K Q T A R K pS T G G K A P R K Q | ▶ | H3 1-19 | S10P | | | | free |
| 18 | A18 | A R T K Q T A R K S pT G G K A P R K Q | ▶ | H3 1-19 | T11P | | | | free |
| 19 | A19 | A R T K Q T A R K S T G G Kac A P R K Q | ▶ | H3 1-19 | K14ac | | | | free |
| 20 | A20 | A Rme2s pT K Q T A R K S T G G K A P R K Q | ▶ | H3 1-19 | R2me2s | T3P | | | free |
| 21 | A21 | A Rme2s T Kme1 Q T A R K S T G G K A P R K Q | ▶ | H3 1-19 | R2me2s | K4me1 | | | free |
| 22 | A22 | A Rme2s T Kme2 Q T A R K S T G G K A P R K Q | ▶ | H3 1-19 | R2me2s | K4me2 | | | free |
| 23 | A23 | A Rme2s T Kme3 Q T A R K S T G G K A P R K Q | ▶ | H3 1-19 | R2me2s | K4me3 | | | free |
| 24 | A24 | A Rme2s T Kac Q T A R K S T G G K A P R K Q | ▶ | H3 1-19 | R2me2s | K4ac | | | free |
| 25 | B 1 | A Rme2a pT K Q T A R K S T G G K A P R K Q | ▶ | H3 1-19 | R2me2a | T3P | | | free |
| 26 | B 2 | A Rme2a T Kme1 Q T A R K S T G G K A P R K Q | ▶ | H3 1-19 | R2me2a | K4me1 | | | free |
| 27 | B 3 | A Rme2a T Kme2 Q T A R K S T G G K A P R K Q | ▶ | H3 1-19 | R2me2a | K4me2 | | | free |
| 28 | B 4 | A Rme2a T Kme3 Q T A R K S T G G K A P R K Q | ▶ | H3 1-19 | R2me2a | K4me3 | | | free |
| 29 | B 5 | A Rme2a T Kac Q T A R K S T G G K A P R K Q | ▶ | H3 1-19 | R2me2a | K4ac | | | free |
| 30 | B 6 | A Cit pT K Q T A R K S T G G K A P R K Q | ▶ | H3 1-19 | R2Citr | T3P | | | free |
| 31 | B 7 | A Cit T Kme1 Q T A R K S T G G K A P R K Q | ▶ | H3 1-19 | R2Citr | K4me1 | | | free |
| 32 | B 8 | A Cit T Kme2 Q T A R K S T G G K A P R K Q | ▶ | H3 1-19 | R2Citr | K4me2 | | | free |
| 33 | B 9 | A Cit T Kme3 Q T A R K S T G G K A P R K Q | ▶ | H3 1-19 | R2Citr | K4me3 | | | free |
| 34 | B10 | A Cit T Kac Q T A R K S T G G K A P R K Q | ▶ | H3 1-19 | R2Citr | K4ac | | | free |
| 35 | B11 | A R pT Kme1 Q T A R K S T G G K A P R K Q | ▶ | H3 1-19 | T3P | K4me1 | | | free |
| 36 | B12 | A R pT Kme2 Q T A R K S T G G K A P R K Q | ▶ | H3 1-19 | T3P | K4me2 | | | free |
| 37 | B13 | A R pT Kme3 Q T A R K S T G G K A P R K Q | ▶ | H3 1-19 | T3P | K4me3 | | | free |
| 38 | B14 | A R pT Kac Q T A R K S T G G K A P R K Q | ▶ | H3 1-19 | T3P | K4ac | | | free |
| 39 | B15 | A Rme2s pT Kme1 Q T A R K S T G G K A P R K Q | ▶ | H3 1-19 | R2me2s | T3P | K4me1 | | free |
| 40 | B16 | A Rme2s pT Kme2 Q T A R K S T G G K A P R K Q | ▶ | H3 1-19 | R2me2s | T3P | K4me2 | | free |
| 41 | B17 | A Rme2s pT Kme3 Q T A R K S T G G K A P R K Q | ▶ | H3 1-19 | R2me2s | T3P | K4me3 | | free |
| 42 | B18 | A Rme2s pT Kac Q T A R K S T G G K A P R K Q | ▶ | H3 1-19 | R2me2s | T3P | K4ac | | free |
| 43 | B19 | A Rme2a pT Kme1 Q T A R K S T G G K A P R K Q | ▶ | H3 1-19 | R2me2a | T3P | K4me1 | | free |
| 44 | B20 | A Rme2a pT Kme2 Q T A R K S T G G K A P R K Q | ▶ | H3 1-19 | R2me2a | T3P | K4me2 | | free |
| 45 | B21 | A Rme2a pT Kme3 Q T A R K S T G G K A P R K Q | ▶ | H3 1-19 | R2me2a | T3P | K4me3 | | free |
| 46 | B22 | A Rme2a pT Kac Q T A R K S T G G K A P R K Q | ▶ | H3 1-19 | R2me2a | T3P | K4ac | | free |
| 47 | B23 | A R T K Q T A Rme2a Kme1 S T G G K A P R K Q | ▶ | H3 1-19 | R8me2s | K9me1 | | | free |
| 48 | B24 | A R T K Q T A Rme2a Kme2 S T G G K A P R K Q | ▶ | H3 1-19 | R8me2s | K9me2 | | | free |
| 49 | C 1 | A R T K Q T A Rme2a Kme3 S T G G K A P R K Q | ▶ | H3 1-19 | R8me2s | K9me3 | | | free |
| 50 | C 2 | A R T K Q T A Rme2a Kac S T G G K A P R K Q | ▶ | H3 1-19 | R8me2s | K9ac | | | free |
| 51 | C 3 | A R T K Q T A Rme2a K pS T G G K A P R K Q | ▶ | H3 1-19 | R8me2s | S10P | | | free |
| 52 | C 4 | A R T K Q T A Rme2a K S pT G G K A P R K Q | ▶ | H3 1-19 | R8me2s | T11P | | | free |
| 53 | C 5 | A R T K Q T A Rme2a Kme1 S T G G K A P R K Q | ▶ | H3 1-19 | R8me2a | K9me1 | | | free |
| 54 | C 6 | A R T K Q T A Rme2a Kme2 S T G G K A P R K Q | ▶ | H3 1-19 | R8me2a | K9me2 | | | free |
| 55 | C 7 | A R T K Q T A Rme2a Kme3 S T G G K A P R K Q | ▶ | H3 1-19 | R8me2a | K9me3 | | | free |
| 56 | C 8 | A R T K Q T A Rme2a Kac S T G G K A P R K Q | ▶ | H3 1-19 | R8me2a | K9ac | | | free |
| 57 | C 9 | A R T K Q T A Rme2a K pS T G G K A P R K Q | ▶ | H3 1-19 | R8me2a | S10P | | | free |
| 58 | C10 | A R T K Q T A Rme2a K S pT G G K A P R K Q | ▶ | H3 1-19 | R8me2a | T11P | | | free |
| 59 | C11 | A R T K Q T A Cit Kme1 S T G G K A P R K Q | ▶ | H3 1-19 | R8Citr | K9me1 | | | free |
| 60 | C12 | A R T K Q T A Cit Kme2 S T G G K A P R K Q | ▶ | H3 1-19 | R8Citr | K9me2 | | | free |
| 61 | C13 | A R T K Q T A Cit Kme3 S T G G K A P R K Q | ▶ | H3 1-19 | R8Citr | K9me3 | | | free |
| 62 | C14 | A R T K Q T A Cit Kac S T G G K A P R K Q | ▶ | H3 1-19 | R8Citr | K9ac | | | free |
| 63 | C15 | A R T K Q T A Cit K pS T G G K A P R K Q | ▶ | H3 1-19 | R8Citr | S10P | | | free |
| 64 | C16 | A R T K Q T A Cit K S pT G G K A P R K Q | ▶ | H3 1-19 | R8Citr | T11P | | | free |
| 65 | C17 | A R T K Q T A R Kme1 pS T G G K A P R K Q | ▶ | H3 1-19 | K9me1 | S10P | | | free |
| 66 | C18 | A R T K Q T A R Kme1 S pT G G K A P R K Q | ▶ | H3 1-19 | K9me1 | T11P | | | free |
| 67 | C19 | A R T K Q T A R Kme1 S T G G Kac A P R K Q | ▶ | H3 1-19 | K9me1 | K14ac | | | free |
| 68 | C20 | A R T K Q T A R Kme2 pS T G G K A P R K Q | ▶ | H3 1-19 | K9me2 | S10P | | | free |
| 69 | C21 | A R T K Q T A R Kme2 S pT G G K A P R K Q | ▶ | H3 1-19 | K9me2 | T11P | | | free |
| 70 | C22 | A R T K Q T A R Kme2 S T G G Kac A P R K Q | ▶ | H3 1-19 | K9me2 | K14ac | | | free |
| 71 | C23 | A R T K Q T A R Kme3 pS T G G K A P R K Q | ▶ | H3 1-19 | K9me3 | S10P | | | free |
| 72 | C24 | A R T K Q T A R Kme3 S pT G G K A P R K Q | ▶ | H3 1-19 | K9me3 | T11P | | | free |
| 73 | D 1 | A R T K Q T A R Kme3 S T G G Kac A P R K Q | ▶ | H3 1-19 | K9me3 | K14ac | | | free |
| 74 | D 2 | A R T K Q T A R Kac pS T G G K A P R K Q | ▶ | H3 1-19 | K9ac | S10P | | | free |
| 75 | D 3 | A R T K Q T A R Kac S pT G G K A P R K Q | ▶ | H3 1-19 | K9ac | T11P | | | free |
| 76 | D 4 | A R T K Q T A R Kac S T G G Kac A P R K Q | ▶ | H3 1-19 | K9ac | K14ac | | | free |
| 77 | D 5 | A R T K Q T A R K pS pT G G K A P R K Q | ▶ | H3 1-19 | S10P | T11P | | | free |
| 78 | D 6 | A R T K Q T A R K pS T G G Kac A P R K Q | ▶ | H3 1-19 | S10P | K14ac | | | free |
| 79 | D 7 | A R T K Q T A R K S pT G G Kac A P R K Q | ▶ | H3 1-19 | T11P | K14ac | | | free |
| 80 | D 8 | A R T K Q T A Rme2s Kme1 pS T G G K A P R K Q | ▶ | H3 1-19 | R8me2s | K9me1 | S10P | | free |
| 81 | D 9 | A R T K Q T A Rme2s Kme2 pS T G G K A P R K Q | ▶ | H3 1-19 | R8me2s | K9me2 | S10P | | free |
| 82 | D10 | A R T K Q T A Rme2s Kme3 pS T G G K A P R K Q | ▶ | H3 1-19 | R8me2s | K9me3 | S10P | | free |
| 83 | D11 | A R T K Q T A Rme2s Kac pS T G G K A P R K Q | ▶ | H3 1-19 | R8me2s | K9ac | S10P | | free |
| 84 | D12 | A R T K Q T A Rme2s Kme1 S pT G G K A P R K Q | ▶ | H3 1-19 | R8me2s | K9me1 | T11P | | free |
| 85 | D13 | A R T K Q T A Rme2s Kme2 S pT G G K A P R K Q | ▶ | H3 1-19 | R8me2s | K9me2 | T11P | | free |
| 86 | D14 | A R T K Q T A Rme2s Kme3 S pT G G K A P R K Q | ▶ | H3 1-19 | R8me2s | K9me3 | T11P | | free |
| 87 | D15 | A R T K Q T A Rme2s Kac S pT G G K A P R K Q | ▶ | H3 1-19 | R8me2s | K9ac | T11P | | free |
| 88 | D16 | A R T K Q T A Rme2a Kme1 pS T G G K A P R K Q | ▶ | H3 1-19 | R8me2a | K9me1 | S10P | | free |
| 89 | D17 | A R T K Q T A Rme2a Kme2 pS T G G K A P R K Q | ▶ | H3 1-19 | R8me2a | K9me2 | S10P | | free |
| 90 | D18 | A R T K Q T A Rme2a Kme3 pS T G G K A P R K Q | ▶ | H3 1-19 | R8me2a | K9me3 | S10P | | free |
| 91 | D19 | A R T K Q T A Rme2a Kac pS T G G K A P R K Q | ▶ | H3 1-19 | R8me2a | K9ac | S10P | | free |

**Supplementary Table 3. A list of modified histones on the MODIFY histone array** Using the commercial histone array specificity factors were determined for arrays probed with OGA-L, HAT domain or OGA-S. These specificity factors shown in supplementary figure 6 (bottom) are plotted for each of the 10 highest specificity factors observed for each of the proteins examined. Only specificity factors higher than 5 were judged to be significant over multiple arrays.

| Name | Product # | 2˚ Antibody | Use in | Storage | manufacturer |
|---|---|---|---|---|---|
| H3 | ab1791 | Rabbit | M, H, C | Aliquoted -20˚ | Abcam |
| H3 S28P | ab5169 | Rabbit | H | Aliquoted -20˚ | Abcam |
| H3 K36Ac | 07-540 | Rabbit | H | -20˚ | Merck Millipore |
| H3 K36Me | ab9048 | Rabbit | M,H | Aliquoted -20˚ | Abcam |
| H3 K36Me2 | ab9049 | Rabbit | M, H, C | Aliquoted -20˚ | Abcam |
| H3 K36Me3 | ab9050 | Rabbit | M, H, C | Aliquoted -20˚ | Abcam |
| GFP | ab290 | Rabbit | entire GFP | Aliquoted -20˚ | Abcam |
| O-GlcNAc | MA1-076 | Mouse | | Aliquoted -20˚ | Thermo Fisher |
| O-GlcNAc | MA1-072 | Mouse | | Aliquoted -20˚ | Thermo Fisher |
| O-GlcNAc (RL2) | ab2739 | Mouse | | Aliquoted -20˚ | Abcam |
| OGA C-term | SAB4200311 | Rabbit | H | Aliquoted -20˚ | Sigma Aldrich |
| OGT (H-300) | sc-32921 | Rabbit | M, H | 4˚ | Santa Cruz |
| N-COAT (H-300) | sc-135093 | Rabbit | M, H | 4˚ | Santa Cruz |
| OGA | sc-135093 | Monoclonal Rabbit | M, H | Aliquoted -20˚ | Santa Cruz |
| OGA | H00010724-MO2 | Mouse | H | -80˚ | Novus Biologicals |
| Anti-6His | His.H8 | mouse | H | -80˚ | Thermo Fisher |
| Anti-Myc | 9E10 | Mouse | H | -80˚ | Thermo-Fisher |
| Anti-c-term | 3D5 | mouse | H | -80˚ | Thermo Fisher |

**Supplementary Table 4: Antibody table.**

| Nucleosome | Manufacturer | Catalog number |
|---|---|---|
| Nucleosome, Recombinant Human, H3 K36me3 dNuc, Biotinylated | EpiCypher | 16-0320 |
| Nucleosome, Recombinant Human, H3 K27me3 dNuc, Biotinylated | EpiCypher | 16-0317 |
| Nucleosome, Recombinant Human, H3.1K27me3,S28phos, Biotinylated | EpiCypher | 16-0397 |
| Nucleosome, Recombinant Human, H4 Tetraacetyl(H4K5, 8, 12, 16 Ac) dNuc, Biotinylated | EpiCypher | 16-0313 |
| Mononucleosomes, Recombinant Human Biotinylated | EpiCypher | 16-0006 |

**Supplementary Table 5: Modified recombinant nucleosomes tested.**
