# Supplementary material for: Human O-GlcNAcase catalytic-stalk dimer anchors flexible histone binding domains
Source: Commun Chem. 2025 Dec 9;9:8. doi: 10.1038/s42004-025-01813-7 (PMC12775002; doi:10.1038/s42004-025-01813-7)
Supplement: Supplementary file 2 — Description of Additional Supplementary Files [file 42004_2025_1813_MOESM2_ESM.pdf]

Description of Additional Supplementary Files:

File: Supplementary Movie

Description: Morph between the cryo-EM model of the Apo OGA-L and the 5m7r crystal structure. Video shows a side and top view of the morph between the two models. OGA catalytic domain is blue, and the stalk is yellow

File: Supplementary Data

Description: Numerical source data file for the graphs and charts has been provided as a Microsoft Excel file
