# Supplementary material for: Human O-GlcNAcase catalytic-stalk dimer anchors flexible histone binding domains
Source: Commun Chem. 2025 Dec 9;9:8. doi: 10.1038/s42004-025-01813-7 (PMC12775002; doi:10.1038/s42004-025-01813-7)
Supplement: Supplementary file 5 — Reporting Summary [file 42004_2025_1813_MOESM5_ESM.pdf]

nature portfolio

Corresponding author(s): John A. Hanover, Jenny E. Hinshaw

Last updated by author(s): 11/07/2025

Reporting Summary

Nature Portfolio wishes to improve the reproducibility of the work that we publish. This form provides structure for consistency and transparency in reporting. For further information on Nature Portfolio policies, see our Editorial Policies and the Editorial Policy Checklist.

Statistics

For all statistical analyses, confirm that the following items are present in the figure legend, table legend, main text, or Methods section.

n/a	Confirmed	
☐	☒	The exact sample size (*n*) for each experimental group/condition, given as a discrete number and unit of measurement
☐	☒	A statement on whether measurements were taken from distinct samples or whether the same sample was measured repeatedly
☐	☒	The statistical test(s) used AND whether they are one- or two-sided<br>*Only common tests should be described solely by name; describe more complex techniques in the Methods section.*
☐	☒	A description of all covariates tested
☐	☒	A description of any assumptions or corrections, such as tests of normality and adjustment for multiple comparisons
☐	☒	A full description of the statistical parameters including central tendency (e.g. means) or other basic estimates (e.g. regression coefficient) AND variation (e.g. standard deviation) or associated estimates of uncertainty (e.g. confidence intervals)
☒	☐	For null hypothesis testing, the test statistic (e.g. *F*, *t*, *r*) with confidence intervals, effect sizes, degrees of freedom and *P* value noted<br>*Give P values as exact values whenever suitable.*
☒	☐	For Bayesian analysis, information on the choice of priors and Markov chain Monte Carlo settings
☒	☐	For hierarchical and complex designs, identification of the appropriate level for tests and full reporting of outcomes
☒	☐	Estimates of effect sizes (e.g. Cohen's *d*, Pearson's *r*), indicating how they were calculated

Our web collection on statistics for biologists contains articles on many of the points above.

Software and code

Policy information about availability of computer code

Data collection	All code used was publicly available. CryoEM data was collected on either a FEI Krios microscope, wtih Gatan K3 direct detectors, using the program Serial EM.
Data analysis	All code used was publicly available. or drift correction of the cryoEM images we used patch motion correction in cryoSPARC. Data processing for final datasets were done in cryoSPARC v4.6.0, which generated the final reconstructions. CTF was corrected in cryoSPARC using patch ctf estimations. Supplemental data processing utilized deepEMhancer. Model refinement was done using the Rosetta v2021.16, Coot v0.9.8.92, EMAN v2.99.66 and Phenix v.1.29.1-4487 software suites, using geometry-based minimization and real-space refinement. The model was first docked into the cryoEM density manually in UCSF Chimera 1.15 and ChimeraX 1.2.5.Histone arrays were analyzed by ImageJ and Array Analyze Software.

For manuscripts utilizing custom algorithms or software that are central to the research but not yet described in published literature, software must be made available to editors and reviewers. We strongly encourage code deposition in a community repository (e.g. GitHub). See the Nature Portfolio guidelines for submitting code & software for further information.

Data

Policy information about availability of data

All manuscripts must include a data availability statement. This statement should provide the following information, where applicable:

- Accession codes, unique identifiers, or web links for publicly available datasets
- A description of any restrictions on data availability
- For clinical datasets or third party data, please ensure that the statement adheres to our policy

Data availability
Structural data supporting findings in this study have been deposited in the Protein Data Bank (PDB) and the Electron Microscopy Data Bank (EMDB). The accession codes of the cryo-EM maps and accompanying atomic models have been provided for: (1) OGA-L Catalytic Dimer (PDB ID: 9EN2, EMDB-49293), 2) A-chain of the OGA-L Catalytic Dimer (PDB ID: 9EN4, EMDB-49294), 3) B-chain of the OGA-L Catalytic Dimer (PDB ID: 9EN5, EMDB-49295), 4) OGA-L Dimer (EMDB-49296), 5) OGA-L Catalytic Dimer, A-chain extra density (EMDB-49297). Antibodies employed in this study can be found in Supplementary Table 2. Additional data compared in this study from the protein databank: Alphafold2 Multimer, PDB ID: 5M7R, PDB ID: 5VVO, PDB ID:5UHK, PDB ID: 5M7S, PDBID:5UN9, PDB ID:5UHL, PDB ID: 5M7T, PDB ID: 5UHO, PDB ID: 8P0L, PDB ID: 9BA8, 9BA9, PDB ID: 1KX5.

Code availability
Custom codes were not central to the conclusions of the paper.

Research involving human participants, their data, or biological material

Policy information about studies with human participants or human data. See also policy information about sex, gender (identity/presentation), and sexual orientation and race, ethnicity and racism.

Reporting on sex and gender	NA
Reporting on race, ethnicity, or other socially relevant groupings	NA
Population characteristics	NA
Recruitment	NA
Ethics oversight	NA

Note that full information on the approval of the study protocol must also be provided in the manuscript.

Field-specific reporting

Please select the one below that is the best fit for your research. If you are not sure, read the appropriate sections before making your selection.

☒ Life sciences ☐ Behavioural & social sciences ☐ Ecological, evolutionary & environmental sciences

For a reference copy of the document with all sections, see nature.com/documents/nr-reporting-summary-flat.pdf

Life sciences study design

All studies must disclose on these points even when the disclosure is negative.

Sample size	All sample sizes are described and replicates were performed including replicate samples on single chips for histone and mononucleosome assays. Our cryoEM reconstructions of the OGA-L catalytic-stalk dimer, OGA-L catalytic-stalk A-Chain, OGA-L catalytic-stalk B-chain, OGA-L catalytic-stalk with extra A-chain density, and OGA-L dimer contained 65,831 particles, 65,831 particles, 65,831 particles, 62,230 particles, and 8,527 particles respectively. These sample sizes were determined through image processing software, and were sufficient to converge the structure calculation to detailed maps that are consistent with crystal structures and functional studies. Furthermore, all Euler angles were well sampled in the reconstructions.
Data exclusions	Only one antibody was excluded from the analysis since it did not show specificity for the epitope tag used. Particles were excluded from the final reconstructions in order to enhance the homogeneity of the final image set, which enabled a higher resolution reconstruction.
Replication	Multiple replicates performed as described in Materials and Methods. For cryoEM, the OGA-L dimer conformation was reproducible. This consistency was necessary in order to accrue sufficient sample to be imaged for cryo-EM.
Randomization	The arrays used include unbiased representation of histones and PTM. Image particles were allocated to different 2D classes based on a maximum likelihood algorithm implemented by the software cryoSPARC. Furthermore, in order to minimize model bias, the initial model was the result of ab-inito reconstruction.

Blinding	The assignment of particles to classes during reconstruction is implemented automatically by the cryoSPARC software, without direct supervision by the investigator.

Behavioural & social sciences study design

All studies must disclose on these points even when the disclosure is negative.

Study description	NA
Research sample	NA
Sampling strategy	NA
Data collection	NA
Timing	NA
Data exclusions	NA
Non-participation	NA
Randomization	NA

Ecological, evolutionary & environmental sciences study design

All studies must disclose on these points even when the disclosure is negative.

Study description	NA
Research sample	NA
Sampling strategy	NA
Data collection	NA
Timing and spatial scale	NA
Data exclusions	NA
Reproducibility	NA
Randomization	NA
Blinding	NA

Did the study involve field work? ☐ Yes ☒ No

Field work, collection and transport

Field conditions	*Describe the study conditions for field work, providing relevant parameters (e.g. temperature, rainfall).*
Location	*State the location of the sampling or experiment, providing relevant parameters (e.g. latitude and longitude, elevation, water depth).*
Access & import/export	*Describe the efforts you have made to access habitats and to collect and import/export your samples in a responsible manner and in compliance with local, national and international laws, noting any permits that were obtained (give the name of the issuing authority, the date of issue, and any identifying information).*
Disturbance	*Describe any disturbance caused by the study and how it was minimized.*

Reporting for specific materials, systems and methods

We require information from authors about some types of materials, experimental systems and methods used in many studies. Here, indicate whether each material, system or method listed is relevant to your study. If you are not sure if a list item applies to your research, read the appropriate section before selecting a response.

Materials & experimental systems

n/a	Involved in the study
[ ]	[x] Antibodies
[ ]	[x] Eukaryotic cell lines
[x]	[ ] Palaeontology and archaeology
[x]	[ ] Animals and other organisms
[x]	[ ] Clinical data
[x]	[ ] Dual use research of concern
[x]	[ ] Plants

Methods

n/a	Involved in the study
[x]	[ ] ChIP-seq
[x]	[ ] Flow cytometry
[x]	[ ] MRI-based neuroimaging

Antibodies

Antibodies used

An antibody table is provided with this material in the manuscript:Name Product # 2˚ Antibody Use in Storage manufacturer
H3 ab1791 Rabbit M, H, C Aliquoted -20 Abcam
H3 S28P ab5169 Rabbit H Aliquoted -20 Abcam
H3 K36Ac 07-540 Rabbit H -20 Merck Millipore
H3 K36Me ab9048 Rabbit M,H Aliquoted -20 Abcam
H3 K36Me2 ab9049 Rabbit M, H, C Aliquoted -20 Abcam
H3 K36Me3 ab9050 Rabbit M, H, C Aliquoted -20 Abcam
GFP ab290 Rabbit entire GFP Aliquoted -20 Abcam
O-GlcNAc MA1-076 Mouse Aliquoted -20 Thermo Fisher
O-GlcNAc MA1-072 Mouse Aliquoted -20 Thermo Fisher
O-GlcNAc (RL2) ab2739 Mouse Aliquoted -20 Abcam
OGA C-term SAB4200311 Rabbit H Aliquoted -20 Sigma Aldrich
OGT (H-300) sc-32921 Rabbit M, H 4˚ Santa Cruz
N-COAT (H-300) sc-135093 Rabbit M, H 4˚ Santa Cruz
OGA sc-135093 Monoclonal Rabbit M, H Aliquoted -20 Santa Cruz
OGA H00010724-MO2 Mouse H -80 Novus Biologicals
Anti-6His His.H8 mouse H -80 Thermo Fisher
Anti-Myc 9E10 Mouse H -80 Thermo-Fisher
Anti-c-term 3D5 mouse H -80 Thermo Fisher

Validation

Antibody validation is included in a table in the manuscript:
Name Product # 2˚ Antibody Use in Storage manufacturer
H3 ab1791 Rabbit M, H, C Aliquoted -20 Abcam
H3 S28P ab5169 Rabbit H Aliquoted -20 Abcam
H3 K36Ac 07-540 Rabbit H -20 Merck Millipore
H3 K36Me ab9048 Rabbit M,H Aliquoted -20 Abcam
H3 K36Me2 ab9049 Rabbit M, H, C Aliquoted -20 Abcam
H3 K36Me3 ab9050 Rabbit M, H, C Aliquoted -20 Abcam
GFP ab290 Rabbit entire GFP Aliquoted -20 Abcam
O-GlcNAc MA1-076 Mouse Aliquoted -20 Thermo Fisher
O-GlcNAc MA1-072 Mouse Aliquoted -20 Thermo Fisher
O-GlcNAc (RL2) ab2739 Mouse Aliquoted -20 Abcam
OGA C-term SAB4200311 Rabbit H Aliquoted -20 Sigma Aldrich
OGT (H-300) sc-32921 Rabbit M, H 4˚ Santa Cruz
N-COAT (H-300) sc-135093 Rabbit M, H 4˚ Santa Cruz
OGA sc-135093 Monoclonal Rabbit M, H Aliquoted -20 Santa Cruz
OGA H00010724-MO2 Mouse H -80 Novus Biologicals
Anti-6His His.H8 mouse H -80 Thermo Fisher
Anti-Myc 9E10 Mouse H -80 Thermo-Fisher
Anti-c-term 3D5 mouse H -80 Thermo Fisher

Eukaryotic cell lines

Policy information about cell lines and Sex and Gender in Research

Cell line source(s)

Mouse Embryonic Fibroblast (MEFs) were generated in our laboratory

Authentication

Cell lines were authenticated

Mycoplasma contamination

Cell line was checked to confirm no micoplasma contamination

Commonly misidentified lines (See ICLAC register)	NA

Palaeontology and Archaeology

Specimen provenance	NA
Specimen deposition	NA
Dating methods	NA

☐ Tick this box to confirm that the raw and calibrated dates are available in the paper or in Supplementary Information.

Ethics oversight	NA

Note that full information on the approval of the study protocol must also be provided in the manuscript.

Animals and other research organisms

Policy information about studies involving animals; ARRIVE guidelines recommended for reporting animal research, and Sex and Gender in Research

Laboratory animals	NA
Wild animals	NA
Reporting on sex	NA
Field-collected samples	NA
Ethics oversight	NA

Note that full information on the approval of the study protocol must also be provided in the manuscript.

Clinical data

Policy information about clinical studies

All manuscripts should comply with the ICMJEguidelines for publication of clinical research and a completedCONSORT checklist must be included with all submissions.

Clinical trial registration	NA
Study protocol	NA
Data collection	NA
Outcomes	NA

Dual use research of concern

Policy information about dual use research of concern

Hazards

Could the accidental, deliberate or reckless misuse of agents or technologies generated in the work, or the application of information presented in the manuscript, pose a threat to:

No	Yes	
☒	☐	Public health
☒	☐	National security
☒	☐	Crops and/or livestock
☒	☐	Ecosystems
☒	☐	Any other significant area

Experiments of concern

Does the work involve any of these experiments of concern:

No	Yes	
☒	☐	Demonstrate how to render a vaccine ineffective
☒	☐	Confer resistance to therapeutically useful antibiotics or antiviral agents
☒	☐	Enhance the virulence of a pathogen or render a nonpathogen virulent
☒	☐	Increase transmissibility of a pathogen
☒	☐	Alter the host range of a pathogen
☒	☐	Enable evasion of diagnostic/detection modalities
☒	☐	Enable the weaponization of a biological agent or toxin
☒	☐	Any other potentially harmful combination of experiments and agents

Plants

Seed stocks	NA
Novel plant genotypes	NA
Authentication	NA

ChIP-seq

Data deposition

☐ Confirm that both raw and final processed data have been deposited in a public database such as GEO.

☐ Confirm that you have deposited or provided access to graph files (e.g. BED files) for the called peaks.

Data access links<br>*May remain private before publication.*	NA
Files in database submission	NA
Genome browser session<br>(e.g. UCSC)	NA

Methodology

Replicates	NA
Sequencing depth	NA
Antibodies	NA
Peak calling parameters	NA
Data quality	NA
Software	NA

Flow Cytometry

Plots

Confirm that:

- [ ] The axis labels state the marker and fluorochrome used (e.g. CD4-FITC).
- [ ] The axis scales are clearly visible. Include numbers along axes only for bottom left plot of group (a 'group' is an analysis of identical markers).
- [ ] All plots are contour plots with outliers or pseudocolor plots.
- [ ] A numerical value for number of cells or percentage (with statistics) is provided.

Methodology

Sample preparation	NA
Instrument	NA
Software	NA
Cell population abundance	NA
Gating strategy	NA

- [ ] Tick this box to confirm that a figure exemplifying the gating strategy is provided in the Supplementary Information.

Magnetic resonance imaging

Experimental design

Design type	NA
Design specifications	NA
Behavioral performance measures	NA

Acquisition

Imaging type(s)	NA
Field strength	NA
Sequence & imaging parameters	NA
Area of acquisition	NA

Diffusion MRI [ ] Used [x] Not used

Preprocessing

Preprocessing software	NA
Normalization	NA
Normalization template	NA
Noise and artifact removal	NA
Volume censoring	NA

Statistical modeling & inference

Model type and settings	NA
Effect(s) tested	NA

Specify type of analysis: [ ] Whole brain [ ] ROI-based [ ] Both

Statistic type for inference

(See Eklund et al. 2016)

NA

Correction

NA

Models & analysis

n/a	Involved in the study
☒	☐ Functional and/or effective connectivity
☒	☐ Graph analysis
☒	☐ Multivariate modeling or predictive analysis

Functional and/or effective connectivity

NA

Graph analysis

NA

Multivariate modeling and predictive analysis

NA
